# Central Role of the Gut Epithelial Barrier in the Pathogenesis of Chronic Intestinal Inflammation: Lessons Learned from Animal Models and Human Genetics

**DOI:** 10.3389/fimmu.2013.00280

**Published:** 2013-09-17

**Authors:** Luca Pastorelli, Carlo De Salvo, Joseph R. Mercado, Maurizio Vecchi, Theresa T. Pizarro

**Affiliations:** ^1^Department of Pathology, Case Western Reserve University School of Medicine, Cleveland, OH, USA; ^2^Department of Biomedical Sciences for Health, University of Milan, Milan, Italy; ^3^Gastroenterology and Digestive Endoscopy Unit, IRCCS Policlinico San Donato, San Donato Milanese, Italy

**Keywords:** intestinal epithelial cells, intestinal barrier function, gut immune homeostasis, innate immunity, Crohn’s disease, ulcerative colitis, inflammatory bowel disease genetics, animal models of intestinal inflammation

## Abstract

The gut mucosa is constantly challenged by a bombardment of foreign antigens and environmental microorganisms. As such, the precise regulation of the intestinal barrier allows the maintenance of mucosal immune homeostasis and prevents the onset of uncontrolled inflammation. In support of this concept, emerging evidence points to defects in components of the epithelial barrier as etiologic factors in the pathogenesis of inflammatory bowel diseases (IBDs). In fact, the integrity of the intestinal barrier relies on different elements, including robust innate immune responses, epithelial paracellular permeability, epithelial cell integrity, as well as the production of mucus. The purpose of this review is to systematically evaluate how alterations in the aforementioned epithelial components can lead to the disruption of intestinal immune homeostasis, and subsequent inflammation. In this regard, the wealth of data from mouse models of intestinal inflammation and human genetics are pivotal in understanding pathogenic pathways, for example, that are initiated from the specific loss of function of a single protein leading to the onset of intestinal disease. On the other hand, several recently proposed therapeutic approaches to treat human IBD are targeted at enhancing different elements of gut barrier function, further supporting a primary role of the epithelium in the pathogenesis of chronic intestinal inflammation and emphasizing the importance of maintaining a healthy and effective intestinal barrier.

## Introduction

The gastrointestinal tract, from the beginning of extrauterine life, is chronically exposed to a huge burden of foreign antigens, various microorganisms, and toxic molecules. Therefore, its ability to act as a barrier against potentially harmful molecules and to defend against pathogenic bacteria is pivotal in maintaining gut immune homeostasis. In fact, evolution has selected different mechanisms by which the gut serves as an effective protective barrier. Of paramount importance is the intestinal epithelium of which intestinal epithelial cells (IECs) are the primary cell type coming into contact with the external environment and act as the host’s first line of the defense against potential harmful stimulants. Despite their non-hematopoietic derivation, IECs also represent a core element of innate immunity within the gut mucosa, displaying a wide array of immune functions. In fact, IECs are able to recognize pathogens through the expression of innate immune receptors, to release anti-microbial molecules, and to secrete cytokines and chemokines that link innate and adaptive immune responses. Moreover, IECs also represent the main structural component of the physical barrier between the luminal microenvironment and host, allowing selective absorption of nutrients and denying entry of noxious molecules and antigens. The intestinal epithelium constitutes the largest exposed surface area of the human body and its permeability is finely regulated by the presence of tight junctions (TJs), large molecular complexes which, together with adherens junctions (AJs), link IECs to each other, and seal the intercellular spaces on the luminal surface, regulating molecule passage through the paracellular spaces. Finally, IECs produce the mucus layer covering the entire length of the gastrointestinal tract, whose role is to further protect the mucosal surface from harmful molecules and bacteria, and reinforce the overall intestinal barrier. As such, any defect in these IEC-specific processes can cause a breakdown in gut barrier and consequently, a disruption of normal mucosal immune homeostasis that can potentially lead to uncontrolled chronic inflammation, such as that observed in inflammatory bowel disease (IBD).

## Defects in Epithelial-Specific Innate Immune Functions Lead to Intestinal Inflammation

Intestinal epithelial cells, located at the interface between the external environment and the internal mucosal immune system, must be able to mount early and appropriate defense responses against various pathogens in order to maintain homeostasis. Central to this process are innate immune receptor molecules, referred to as pattern-recognition receptors (PRRs), whose function is to sense highly conserved structures or pathogen-associated molecular patterns (PAMPs) present among several different pathogens (1). Archetypal molecules belonging to PRRs are the toll-like receptors (TLRs), which are type I integral transmembrane glycoproteins expressed by several types of cells, including IECs. The TLR family consists of at least 13 members with slightly different structures that recognize, through an extracellular domain containing large leucine-rich repeats, different PAMPs, such as lipopolysaccharide (LPS), peptidoglycan (PGN), muramyl dipeptide (MDP), lipoteichoic acids (LTAs), and bacterial DNA (2). Recognition of each TLR-specific PAMP initiates downstream signaling through two different pathways: via the myeloid differentiating factor 88 (MyD88) pathway and via an alternative “MyD88-independent” pathway, both of which lead to activation of NF-κB, triggering of other innate immune responses, production of cytokines and chemokines, and finally, recruitment of the adaptive immune system (2). Interestingly, the MyD88 pathway is activated by a cytoplasmic domain similar to the interleukin-1 receptor (IL-1R) (3), and both TLR and IL-1R stimulation leads to NF-κB activation (4).

### Genetically engineered models affecting epithelial innate responses

**Figure 1:**
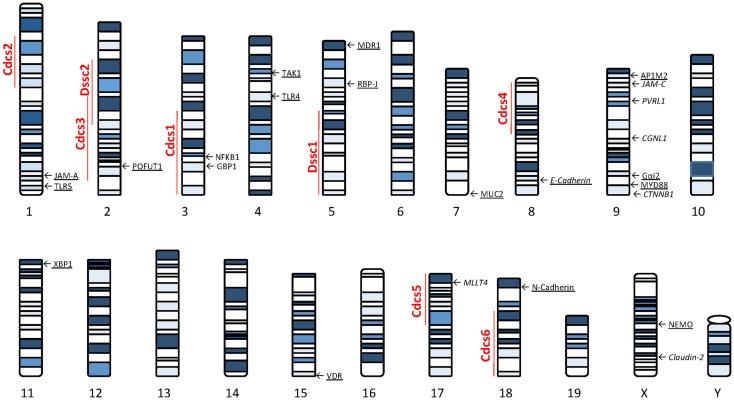
**Murine loci and genes associated with gut inflammation that are potentially related to intestinal epithelial barrier dysfunction**. Colitis susceptibility loci, Cdcs, and Dssc, are identified by red bold font. Genes potentially involved in the epithelial barrier defect characteristic of SAMP mice are italicized. Genes deleted in mouse models of intestinal inflammation that affect epithelial function are underscored. The potential role of each gene in the pathogenesis of epithelial dysfunction associated with chronic intestinal inflammation is discussed within the text. Cdcs, cytokine deficiency-induced colitis susceptibility; Dssc, DSS colitis locus.

Several lines of evidence using genetically manipulated mouse models suggest that deletion/dysregulation of genes and specific chromosomal loci associated with epithelial barrier function can lead to chronic intestinal inflammation (Figure 1). In fact, epithelial barrier defects are clearly present in most animal models of IBD (summarized in Table 1), which have become seminal tools in understanding normal epithelial physiology as well as the role of IECs in the development of gut inflammation.

**Table 1:** **Inflammatory bowel disease animal models with primary defects of intestinal epithelial origin**.

Animal model	Disease location/phenotype of inflammation	Histologic features	Identified gene(s) involved	Epithelial-specific dysfunction
**CHEMICALLY INDUCED MODELS**				
DSS-induced colitis (57)	Superficial colitis	Superficial ulcerations	Loci increasing susceptibility to the induced model found on Chr5 and Chr2	Chemical destruction of mucosal barrier with consequent increase in luminal bacterial translocation
		Infiltration of acute inflammatory cells	
		Crypt distortion	
		Loss of goblet cells	
TNBS-induced colitis (61)	Transmural colitis	Ulceration	N/A	Ethanol-induced destruction of mucosal barrier facilitating hapten penetration and contact with underlying mucosal immune system
		Infiltration of acute/chronic inflammatory cells	
		Crypt distortion	
		Loss of goblet cells	
**GENETICALLY ENGINEERED MODELS AFFECTING EPITHELIAL BARRIER INTEGRITY**				
Mdr1a knockout (103)	Transmural colitis	Colonic thickening with crypt hyperplasia	Mdr1a deletion	Increased basal colonic ion transport
		Focal ulcerations		Dysregulated epithelial cell growth
		Crypt abscesses Leukocyte infiltration Increased number of granulocytes		Increased permeability (dependent on bacterial colonization
				Decreased phosphorylation of TJ proteins (ZO-1 and occludin)
Dominant negative N-cadherin transgenic (62)	Patchy foci of ileal inflammation	Cryptitis and crypt abscesses Epithelial hyperplasia Presence of lymphoid aggregates	Dominant negative N-cadherin expression in small intestinal IEC	Breakdown of intestinal epithelial apical junctional complexes
				Aberrant cell migration, proliferation, and apoptosis in small intestinal crypts
Gai2 knockout (64)	Superficial pancolitis; increased severity in distal colon	Cryptitis and crypt abscesses with crypt distortion	Gai2 deletion	Possible impairment of epithelial TJ assembly
		Mucosal PMN infiltrates	
JAM-A knockout (66)	Colitis	Normal epithelial architecture Increased PMN infiltration Formation of large lymphoid aggregates	JAM-A deletion	Impaired TJ structure and consequent increase in epithelial permeability
				Increase in the TJ proteins, claudin-10 and -15
**GENETICALLY ENGINEERED MODELS AFFECTING EPITHELIAL INNATE RESPONSES**				
TLR5 knockout (5)	Colitis; 10% incidence of rectal prolapse	Mononuclear infiltrates Epithelial hyperplasia Focal crypt loss Goblet cell depletion	TLR5 deletion	Increased epithelial permeability (secondary to inflammation)
				Ineffective bacterial clearance
NEMO^IEC-KO^ (23)	Pancolitis	Mucosal thickening Enlarged crypts Loss of goblet cells Extensive epithelial destruction Marked infiltration of mononuclear cells in mucosa/submucosa	IEC-specific inhibition of NF-κB via conditional ablation of NEMO (IKKg)	Colonic epithelial cell apoptosis (via increased sensitivity to TNF)
				Impaired expression of anti-microbial peptides
				Dysregulated epithelial barrier integrity
TAK1^IEC-KO^ (24)	Enterocolitis	Complete disruption of small intestine structure	IEC specific conditional ablation of TAK1	Colonic epithelial cell apoptosis (via increased sensitivity to TNF)
		Less severe alterations of colonic tissue		Impaired innate immune response
**GENETICALLY ENGINEERED MODELS AFFECTING EPITHELIAL CELL INTEGRITY AND MUCUS PRODUCTION**				
XBP1^IEC-KO^ (100)	Focal non-granulomatous enteritis	Absence of Paneth cells Loss of goblet cells Lamina propria mononuclear infiltrate Crypt abscesses Mucosal ulcerations Villus shortening with a reduction of villus:crypt ratio	IEC specific conditional ablation of XBP1	Impaired innate immune response due to Paneth cell loss by apoptosis
				Endoplasmic reticulum (ER) stress secondary to the lack of XBP1
				Increased proinflammatory signaling due to increased JNK/SAPK activation secondary to the lack of XBP1
AP1M2 knockout (98)	Transmural colitis	Epithelial hyperplasia	Epithelia-specific membrane trafficking factor AP-1B deficiency induced via AP1M2 deletion	Loss of IEC polarity
		Crypt distorsion Loss of goblet cells Mucosal and submucosal inflammatory infiltrate		Impaired epithelial production of anti-microbial peptides
				Defective luminal transport of secretory IgA
**GENETICALLY ENGINEERED MODELS AFFECTING EPITHELIAL CELL INTEGRITY AND MUCUS PRODUCTION**				
RBP-J^IEC-KO^ (99)	Colitis; rectal prolapse	Goblet cell hyperplasia	IEC-specific impairment of Notch signaling via conditional ablation of RBP-J	Retarded IEC turnover
		Aberrant accumulation of mucus under the tunica serosa Neutrophilic infiltrate		Increased epithelial permeability
				Impaired epithelial defense against bacteria
MUC2 knockout (123)	Superficial colitis; more severe in the distal colon	Complete lack of goblet cells	MUC2 deletion	Altered bacterial stimulation of IECs due to a diminished mucus layer
		Crypt hyperplasia	
		Flattening of the epithelial layer and superficial erosions	
		Mild inflammatory infiltration	
		Lamina propria distorsion	
MUC2 mutant (Winnie and Eeyore strains) (124)	Superficial colitis; more severe in the distal colon	Focal epithelial erosions Crypt elongation	MUC2 missense mutations	Altered bacterial stimulation of IECs due to a diminished mucus layer
				Increased endoplasmic reticulum (ER) stress due to mutated MUC2 protein misfolding and accumulation in ER
		Neutrophilic infiltrate	
		Crypt abscesses	
		Goblet cell loss	
POFUT1^IEC-KO^ (128)	Enterocolitis	Crypt hyperplasia	IEC specific conditional ablation of POFUT1	Notch signaling impairment with consequent goblet cell hyperplasia and mucus hypersecretion, leading to associated gut microbiota alterations
		Dilated and mucus filled crypts	
		Hyperplasia of Paneth cells and enteroendocrine cells	
		Inflammatory infiltrate of the lamina propria	
		Crypt abscesses	
		Transmural inflammation	
**SPONTANEOUS MODELS**				
SAMP1/YitFc (74)	Segmental, discontinuous, transmural ileitis; increased severity in the terminal ileum with 2–3% incidence of perianal disease	Villous blunting/crypt hypertrophy Paneth cell/goblet cell hyperplasia PMN/mononuclear cell infiltration in lamina propria and submucosa Aphthous inflammatory lesions Granuloma formation Cryptitis/crypt microabscesses Basal plasmacytosis	Multigenic etiology; susceptibility found on Chr6, Chr8, Chr9, and ChrX	Primary non-hematopoietic (i.e., epithelial) dysfunction
				Increased epithelial permeability independent of commensal bacterial colonization
				Altered TJ protein expression (increase in claudin-2, decrease in occludin)
				Dysregulated epithelial innate responses
C3H/HeJBir (7)	Colitis; primary localization in the cecum	Acute and chronic inflammatory infiltrate	Multigenic etiology; susceptibility found on Chr3, Chr1, Chr2, Chr8, Chr17, and Chr18	Dysregulated epithelial innate responses
		Crypt abscesses		Ineffective bacterial clearance
		Ulcerations Regenerative hyperplasia		Hyper-responsiveness to *flagellin* stimulation

Toll-like receptor-bearing IECs are of critical importance for organizing the first line defense against pathogenic microorganisms and in maintaining normal barrier function. For example, the development of spontaneous intestinal inflammation has been reported in *TLR5* knockout (KO) mice, with around 35–40% of these mice presenting with colitis and exhibiting areas of extensive mononuclear infiltration, epithelial hyperplasia, and focal epithelial crypt destruction (5). An increase in intestinal permeability was also noted in this model, even though it appeared to be secondary to the inflammatory process and not the triggering event. Instead, the primary defect leading to colitis in these mice is speculated to be the waning ability to clear bacteria due to an inherent defect in innate immune responses. Thus, the lack of TLR5 promotes an increase in colonic bacterial burden, and this process may enhance the activation of other proinflammatory pathways. In fact, the absence of colonic inflammation in *TLR4/5* and *IL-1R/TLR5* double KO mouse strains (5, 6) strongly suggests that activation of other Toll/IL-1 receptor pathways, such as TLR4 and IL-1R, is essential for the onset of disease.

The importance of TLR5 signaling in the development of spontaneous gut inflammation has also been brought to light using the spontaneous C3H/HeJBir model of colitis as well as studies in IBD patients, which suggest a central role for TLR5 and bacterial flagellin, its natural ligand, in the pathogenesis of Crohn’s disease (CD), one of the major forms of IBD. The colitis characteristic of C3H/HeJBir mice is primarily localized to the cecum and resolves by 3 months of age (7). Interaction with the commensal bacterial flora is important in this model, as innate responses to TLR ligands are impaired compared to the colitis-resistant C57BL/6 strain (8), with the major class of antigens identified as commensal bacterial flagellins, recognized by TLR5 (9). In fact, serum IgG anti-flagellin antibodies have been identified in three different mouse models and in approximately 50% of CD patients evaluated, but not in either UC patients or controls (9). In addition, flagellin-reactive Th1 cells isolated from C3H/HeJBir mice have the ability to induce colitis upon transfer to naïve SCID recipients. Analysis of quantitative trait locus mapping of C3H/HeJBir mice backcrossed with IL-10 KO mice identified several potential colitogenic loci on chromosome 3, 1, 2, 8, 17, and 18, named, respectively, cytokine deficiency-induced colitis susceptibility 1–6 (*Cdcs1–6*) (10). The strongest association with the colitic phenotype was seen for *Cdcs1*, which includes two attractive candidate genes: encoding nuclear factor kappa B subunit 1 (*Nf*κ*b1*), and encoding guanylate binding protein 1 (*Gbp1*) (8). Quite remarkably, both of these genes encode pivotal proteins in TLR downstream signaling, corroborating data on the impairment of TLR5. Although a direct link to epithelial dysfunction has not been made to the colitis phenotype, these data suggest that C3H/HeJBir mice exhibit defects in TLR5-dependent host-microflora interactions, resulting in increased T cell responses to bacterial antigens (i.e., flagellin).

Activation of the TLR5 pathway also appears to be the mechanism by which adherent-invasive *Escherichia coli* (AIEC) exacerbates inflammation in dextran sulfate sodium (DSS)-induced colitis (11). In these studies, BALBc/J mice treated with DSS and orally challenged with LF82, the reference strain for AIEC that has the ability to adhere to and invade IECs (12) and notably colonizes the inflamed mucosa of ileal CD patients (13), worsened the severity of colitis and induced a sevenfold increase in colonic tissue levels of TLR5 compared to mice infected with a mutated strain of LF82 that lacks the *fli*C gene encoding flagellin (11). These data further support a central role of TLR5 activation in bacterial-host interactions that drive chronic intestinal inflammation. Moreover, a dominant-negative *TLR5* polymorphism, which has been shown to dampen adaptive immune responses to flagellin, appears to reduce the production of IgG against flagellin and to be protective against the development of CD in a Jewish population, suggesting that mucosal immune responses to flagellin promote pathogenic responses in CD (14).

Aside from TLR5, other TLRs, such as TLR4, also appear to play a role in gut mucosal immune homeostasis and in regulating epithelial barrier function against invasive bacteria. *TLR4* and *MyD88* KO mice both develop more severe colitis induced by DSS compared to wildtype (WT) controls, with increased bacterial translocation, shown by the greater positivity of mesenteric lymph node cultures for *E. coli* and *Pseudomonas fluorescence* (15). Remarkably, the analysis of intestinal mucosa from IBD patients has shown a strong upregulation of TLR4 that is normally not expressed in healthy individuals (16), while genetic association studies have linked carriage of the *TLR4* Asp299Gly polymorphism, which has been reported to impair LPS sensing, with IBD susceptibility in different patient populations (17). The prevalence of other TLR genetic polymorphisms has been reported in IBD. TLR1, -2, and -6 non-synonymous polymorphisms have also been shown to be associated with UC and CD colonic disease extension (18). While mechanistic studies have not been reported for TLR1 and -6, *TLR2* KO mice have been shown to be more susceptible to DSS colitis than WT controls (19). These mice display an increase in IEC apoptosis that is secondary to defective goblet cell production of trefoil factor 3 (TFF3), a peptide with anti-apoptotic functions that also enhances mucosal healing (20). Similarly, impairment of TLR9 may also promote the development of intestinal inflammation. Indeed, IECs constitutively expressing TLR9 release potent amounts of the proinflammatory cytokines, TNF, and IL-8, in response to CpG DNA, TLR9’s natural ligand (21). Nonetheless, while a *TLR9* polymorphism has been reported to be associated with CD (22), more experimental data are warranted to clarify the role of TLR9- and other TLR-dependent pathways in the pathogenesis of IBD.

Downstream of TLR signaling, NF-κB activation occurs that results in the initiation of a proinflammatory cascade. Increasing evidence suggests that the NF-κB pathway plays a critical role in regulating epithelial innate responses and maintaining gut homeostasis. This is best illustrated by the *NEMO* KO model in which IEC-specific inhibition of NF-κB, through conditional ablation of NEMO, results in the generation of spontaneous pancolitis (23). In these mutant mice, specific IEC deletion of NF-κB specifically resulted in apoptosis of colonic epithelial cells, impaired expression of anti-microbial peptides, and increased translocation of bacteria into the gut mucosa. In addition, deficiency of the gene encoding MyD88, positioned upstream of NEMO in the NF-κB signaling cascade, prevented colitis and demonstrates that TLR activation by the gut microbiota is essential for disease pathogenesis in this model (23). Similarly, TGF-β-activated kinase 1 (TAK1) is an essential intermediate of innate signaling pathways, and its expression also leads to downstream NF-κB activation. Specific deletion of TAK1 in IECs results in death on postnatal Day 1 in mutant mice, due to severe intestinal bleeding, while TAK1 knockdown in 4-week-old mice leads to the onset of intestinal inflammation, characterized by a complete loss of small intestinal architecture and a marked increase in IEC apoptosis within the crypts of both the ileum and colon (24). In this model however, impairment of innate immunity due to ineffective downstream TLR signaling is not the only mechanism proposed to induce the aforementioned gut pathologies. In fact, double mutant mice, bearing both the intestinal epithelium-specific *TAK1* deletion and the tumor necrosis factor receptor 1 (*TNFR1*) deletion develop less severe gut inflammation and IEC apoptosis, suggesting that TAK1 confers IEC resistance toward TNF-mediated apoptosis during the inflammatory process (24). Taken together, these data indicate that NF-κB not only serves as a master regulator of proinflammatory cytokines, but also functions to control epithelial barrier integrity and interactions between the mucosal immune system and the gut microflora. The role, however, of the TLR/NF-κB pathway in the development of IBD is complex and may be cell-specific in its overall contribution to disease pathogenesis. In fact, whereas TLR/NF-κB engagement on IECs appears to be mostly protective, the activation of the same pathway in cells participating to adaptive immunity is more likely to contribute to intestinal inflammation.

### IBD susceptibility genes associated with epithelial innate immune functions

Emerging evidence suggests that human genetic studies investigating the pathogenesis of IBD strongly corroborate the hypothesis of a fundamental influence of innate immunity in maintaining gut mucosal immune homeostasis. In fact, apart from genes associated with the TLR pathway, the most convincing genetic data linking dysregulated innate immune responses with IBD centers around genes of the caspase recruitment domain/nucleotide-binding oligomerization domain (*CARD/NOD*) family, as well as autophagy-related genes.

*CARD15/NOD2* was the first CD susceptibility gene identified in 2001 (25, 26); the discovery of its association to CD ignited interest in the potential mechanistic defects of innate immunity in the pathogenesis of IBD. *CARD15/NOD2* is located on chromosome 16 and encodes a cytoplasmic protein constitutively expressed in myeloid cells (27), IECs, and Paneth cells of the small bowel (28). The CARD15/NOD2 protein is a PRR involved in recognition of the bacterial cell wall component, MDP (29, 30). Similar to other PRRs, sensing of MDP by CARD15/NOD2 triggers NF-kB activation and subsequent expression of proinflammatory cytokines, including TNF, IL-1, IL-6, IL-8, and IL-18 (27, 30, 31). CARD15/NOD2 is also critical for the release of α-defensins (HD5 and HD6) from Paneth cells (32–34); defensins are anti-microbial peptides synthesized by epithelial cells from the skin, urogenital tract, intestine, and lung (35), whose role is to protect mucosal surfaces by killing pathogens (33, 34). Polymorphisms of *TCF-4*, a transcription factor involved in Paneth cell differentiation and HD5 and HD6 expression, have been reported to be associated with ileal CD (36). On the same line, ileal CD patients carrying *CARD15/NOD2* disease-associated variants display impaired mucosal expression of α-defensins (34, 37), and reduced ability of colonic IEC to excrete β-defensins, in particular human β-defensin 2, in response to MDP (38). The defect in defensin release and IEC response to bacterial challenge may represent a pivotal event in the onset of IBD. In fact, Paneth cells capable of producing high levels of IL-17 if adequately stimulated with TNF have been described (39), that may occur as a result of defective bacterial killing and instead, activation by penetrating pathogens. The IL-23/IL-17 axis has been shown to be involved in autoimmune inflammation, both in humans and animal models (40–42), including IBD (43). Thus, bacterial clearance promoted by CARD15/NOD2 activation appears to be mandatory for maintaining intestinal epithelial barrier function. Nonetheless, other studies suggest that bacterial killing may not be the only mechanism by which CARD15/NOD2 enhances epithelial barrier function. In fact, several studies showed that *CARD15/NOD2* variants are associated with increased intestinal permeability in CD patients and their relatives (34, 44–46); however, the precise mechanism(s) linking a PRR defect and increased intestinal permeability are still largely unknown. Finally, another member of the *CARD/NOD* family, *CARD4/NOD1* that encodes an intracellular receptor with the ability to recognize a unique tripeptide motif (diaminopimelic acid) found in Gram-negative bacterial PGN (47), has been proposed as an IBD susceptibility gene, since three polymorphic variants have been reported to be associated with IBD in two different Caucasian populations (48), adding further interest to the PPR pathways.

Although less developed than the evidence supporting a role for CARD15/NOD2 in the pathogenesis of IBD, recent genetic findings have focused on the importance of another branch of innate immunity, that is autophagy, in the regulation of intestinal inflammation. Indeed, large genome-wide association studies have identified two autophagy-related genes, autophagy-related 16-like 1 (*ATG16L1*) (49) and immunity-related GTPase family M (*IRGM*) (50) on chromosomes 2 and 5, respectively, as CD susceptibility genes. Autophagy is a intracellular process through which cells rearrange their cytoplasm and organelles by means of lysosomal digestion (51), and is considered a response of innate immunity as it represents a major mechanism of defense against intracellular pathogens, such as *Salmonella* or *Mycobacterium* species (49, 52, 53). In fact, functional studies have shown that *ATG16L1* knockdown in IEC lines impairs the clearance *S. typhimurium* infection (49). In addition, since cells undergo a structural de-arrangement during the autophagic process, autophagy has the potential to alter overall epithelial integrity (51). Interestingly, mice that are genetically engineered to under-express the ATG16L1 protein display profound alterations in Paneth cell morphology and function (54). Although these mice do not develop spontaneous gut inflammation, they show a lack of lysozymes in the intestinal mucus, hyperactivation of proinflammatory pathways, and the production of adipokines and acute phase reactants (54).

**Figure 2:**
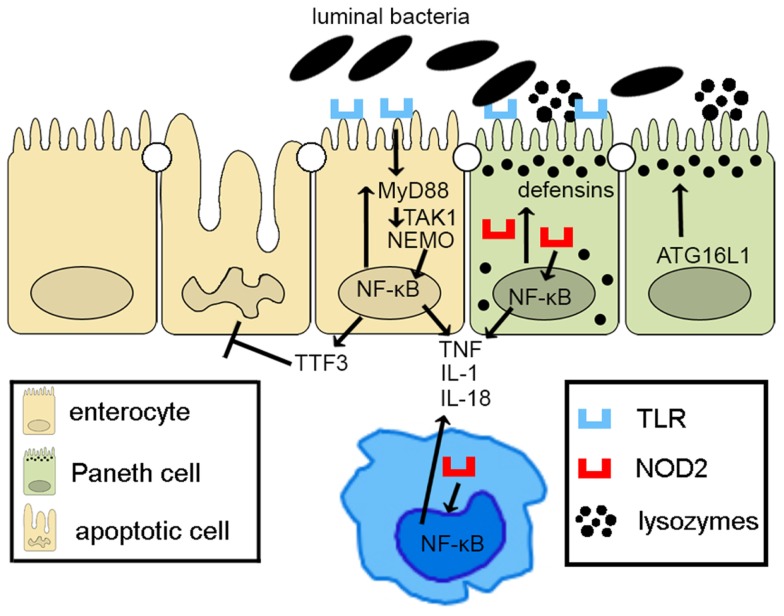
**Epithelial innate immune function is a key factor in maintaining gut homeostasis**. IECs express PRRs, such as TLRs and NOD-like receptors, whose signaling activates NF-κB, leading to reinforcement of the epithelial barrier through release of anti-microbial peptides (i.e., defensins) and paracellular secretion of proinflammatory cytokines (e.g., TNF, IL-1, and IL-18) that enhance mucosal defense to bacterial penetration and the production of trophic factors, such as intestinal TFF3 that can block IEC apoptosis. Autophagy, perhaps due to ATG16L1, also contributes to the effectiveness of the epithelial barrier, controlling intracellular pathogens, and inducing lysozyme production. Breakdown of PRR/NF-κB signaling pathways via critical components, including MyD88, TAK1, and NEMO, facilitates penetrance of luminal microorganisms, triggering an exaggerated adaptive immune response. Similarly, defects in autophagy lead to less effective bacterial clearance and production of proinflammatory molecules, such as adipokines and acute phase reactants from Paneth cells.

Taken together, epithelial innate immune function, including appropriate activation of PRR pathways and of autophagy processes, plays a central role in the overall maintenance of intestinal immune homeostasis. Defects in epithelial innate function can result in dysregulated mucosal immune responses that lead to chronic intestinal inflammation and IBD (Figure 2). As such, IECs embody much more than the mere lining of the gut lumen, but represent the first line of host defense, controlling penetration, and invasion of pathogens, which is critical in limiting adaptive immune activation.

## Defects in Epithelial Barrier Function Lead to Intestinal Inflammation

In addition to the central role of IECs in maintaining mucosal barrier function through early activation of innate immune responses, the intestinal epithelium also constitutes an impermeable layer that has the ability to selectively absorb what is necessary to sustain the organism, while denying passage of other pathogenic and noxious molecules. Disruption of this selective physical barrier, resulting in uncontrolled or dysregulated gut epithelial permeability can induce an overactive mucosal immune response and chronic intestinal inflammation. In fact, since 1972, when Shorter et al. postulated that a primary defect in intestinal permeability might lead to the development of persistent inflammation in the gut, as in the case of IBD (55), a growing body of evidence has supported this theory. Central to this hypothesis, several studies have reported ultrastructural changes in IEC junctional complexes, inducing dramatic changes in gut permeability in both animal models of intestinal inflammation and IBD patients.

### Role of tight junctions in intestinal inflammation

Tight junctions are pivotal in regulating intestinal permeability and the diffusion of ions and molecules across the epithelial luminal surface. TJs consist of at least 50 different membrane-associated proteins located between apical and lateral regions of polarized epithelial cells that link neighboring cells and regulate molecular flow through intercellular spaces (56). TJ proteins include: (1) integral membrane proteins, such as junctional adhesion molecules, claudins, and occludins, which extend into the intercellular spaces and function as a gate, (2) cytoplasmic cytoskeletal linker proteins, such as cingulin, zona occludens-1, -2, -3 (ZO-1, -2, -3), which anchor membrane proteins to the cytoskeleton, and (3) a number of signaling proteins that can activate various downstream cascades, act as transcription factors, and serve as cell cycle regulators (56). Several lines of evidence support the concept that a direct link exists between TJ protein impairment and intestinal inflammation, with the large majority of data generated from animal models of intestinal inflammation.

### Impaired gut permeability in animal models of intestinal inflammation

Chemically induced colitis likely represents the most highly utilized animal models to induce intestinal inflammation. A prototypic example is colitis induced by DSS, which is fed to directly damage the colonic epithelium, resulting in disruption of barrier integrity and a subsequent increase in luminal antigen/bacterial translocation to the underlying components of the gut mucosal immune system (57). In fact, mice exposed to DSS develop an increase in intestinal permeability before the onset of colonic inflammation. Moreover, several changes in TJ assembly occur during the pre-inflammatory stage of this model, such as complete loss of ZO-1 expression and a doubling of occludin-1 expression in colonic epithelia (58). The ensuing inflammation is acute in nature, primarily consisting of neutrophil and macrophage infiltration and expression of associated cytokines. The sensitivity to DSS challenge differs in various mouse strains (e.g., C3H/HeJ mice display increased susceptibility compared to C57BL/6J mice), while genetic studies have identified quantitative trait loci conferring susceptibility to the development of DSS-induced colonic inflammation. These loci, named DSS colitis 1 and 2 (*Dssc1* and *2*), are located on chromosomes 5 and 2, respectively, but further investigation is needed to evaluate the actual susceptibility genes within these loci (59). As such, although a viable model to investigate the process of epithelial damage/repair and the subsequent acute inflammatory events, it should also be noted that acute DSS colitis does not require the presence of T and B cells (60), and therefore, does not represent an appropriate model when investigating more chronic and adaptive immune responses related to IBD pathogenesis.

Similarly, trinitrobenzene sulfonic acid (TNBS)-induced colitis is an alternative chemically induced model in which an ethanol solvent is first administered that permeabilizes the epithelium, followed by TNBS that functions as a sensitizing hapten driving cellular immune responses toward a Th1 polarized phenotype (61). Despite the artificial nature for initiating gut inflammation, chemically induced models of colitis highlight the importance of epithelial barrier disruption, which is likely the primary event leading to the development of colonic inflammation characteristic of these models.

Interestingly, while several genetically engineered mouse models have been commonly challenged with either DSS and/or TNBS to assess their susceptibility toward a colitic phenotype, only a few of these mutant mice with deletion or transgenic expression of genes related to specific junctional complexes develop colitis spontaneously and without further manipulation. For example, mice genetically engineered to express a dominant negative N-cadherin specifically in small intestinal IECs develop a spontaneous IBD phenotype resembling CD (62). Cadherins, together with catenins, are the main constituent of the AJs, cell-to-cell adhesion structures, and are essential for normal gut development. N-cadherin is a transmembrane molecule that regulates calcium-dependent intercellular adhesion and relies on its association with the actin cytoskeleton. In these mice, altered expression of N-cadherin interferes with E-cadherin and leads to ruptures in the epithelial monolayer and to the generation of patchy inflammatory lesions. N-cadherin mutant mice also demonstrate aberrant epithelial proliferation, migration, and programed cell death in small intestinal crypts that eventually lead to adenoma formation. In support of these data, mice conditionally knocked-out for epithelial p120-catenin, a direct cytoplasmic regulator of E-cadherin expression and function, show a phenotype similar to dominant negative N-cadherin mice (63).

Another IBD model that results from a primary epithelial permeability defect is the *G*α*i2* KO mouse strain that develops a pancolitis at 8–12 weeks of age, and for which early indications point to a defective epithelial barrier that occurs prior to histologic inflammation (64). Gαi2 is an inhibitory isoform of G protein subunit α found in IEC as well as lymphocytes that plays an important role in regulating signal transduction through adenylate cyclase; this subunit is also critical in regulating epithelial permeability. In fact, it has been shown that Gαi2 overexpression in IEC monolayers induces TJ assembly, increasing transepithelial electrical resistance (TER) (65). Therefore, a possible impairment of TJ assembly may be responsible for decreased barrier integrity leading to superficial colitis, which is most severe in the distal colon of this mouse strain (64).

Deletion of the *JAM-A* gene, encoding a transmembrane TJ protein, has generated a unique model of intestinal inflammation. *JAM-A* deficient mice display increased colonic epithelial permeability in homozygous mutants that corresponds to an increase in claudin-10 and -15 (66). While the colonic mucosa has normal epithelial architecture, increased polymorphonuclear infiltration, and formation of large lymphoid aggregates are observed in *JAM-A* KO mice that are absent in WT controls (66). JAM-A is also localized to TJs of endothelial cells as well as on the surface of leukocytes, serving as a critical protein in mediating leukocyte migration (67). However, when mice with specific inactivation of *JAM-A* in endothelial and hematopoietic cells (*Tie-2-Cre-JAM-A^−^*^/^^−^mice) (68) were treated with DSS, resulting colonic inflammation was comparable to controls and much less severe than in *JAM-A* KO mice, strengthening the hypothesis of a primary defect of epithelial origin that leads to colitis (69). Interesting, in the same study, reduced levels of JAM-A expression were detected in inflamed tissues from IBD patients compared to controls (69), suggesting that JAM-A may also play an important role in human disease. However, recent studies in *JAM-A* KO mice have also shown that dysregulation in adaptive immunity also plays a role in the development of the colitis phenotype since the lack of T and B cells and, more prominently the absence of CD4^+^ T cells, increases the severity of intestinal inflammation in these mice (70).

Indeed, the phenotype displayed by the three aforementioned strains of genetically modified mice suggests a primary involvement of epithelial barrier dysfunction in the pathogenesis of intestinal inflammation. However, it should be pointed out that, at present, no other mouse strain in which assembly of the intestinal epithelial junctional structure has been altered, develops any significant signs of intestinal inflammation. As an example, even though occludin KO mice exhibit pronounced morphologic alterations within the gastric mucosa, such as mucus cell hyperplasia and complete loss of parietal cells, no evidence of inflammation has been detected in both the stomach as well as along the entire length of the gut (71). Quite surprisingly, the absence of occludin in these mice does not seem to affect intestinal permeability, which appears to be comparable to that observed in WT littermates (71). Similarly, claudin-15 deficient mice display an enhanced proliferation of intestinal crypt cells, resulting in an overt megaintestine phenotype in the upper small bowel, but neither gut inflammation nor increased epithelial paracellular permeability are observed in these mice, despite a decrease in the number of TJ strands within the distal jejunum and without a compensatory increase in the synthesis of other claudins (72). These observations suggest that both occludin and claudin-15 may be involved in epithelial differentiation/growth regulation, but likely do not play a critical role in the regulation of intestinal epithelial permeability. Similarly, transgenic mice expressing constantly active myosin light chain kinase (CA-MLCK) show a marked increase in intestinal permeability and overexpression of IFNγ, TNF, and IL-4, but no overt histologic signs of intestinal inflammation. MLCK is a kinase that, upon TNF stimulation, phosphorylates myosin II regulatory light chain, leading to TJ rearrangement, and reduction of intestinal barrier function. Interestingly, CD4^+^CD45RB^hi^ adoptive transfer into CA-MLCKRAG1^−/−^ mice causes a much more severe colitis, with an earlier onset, compared to transfer into RAG^−/−^ mice (73).

Taken together, these data suggest that, perhaps, deletion, or deficiency of a singly TJ protein may not be sufficient to disrupt intestinal barrier function alone, and may require a particular combination or a greater number of TJs to be altered and/or dysregulated before overt gut inflammation is observed. Alternatively, the possibility exists that dysfunction of TJ assembly or TJs themselves may not be causal for the generation of chronic intestinal inflammation.

The aforementioned animal models, although extremely useful in investigating specific molecular mechanisms in the pathogenesis of IBD, do not fully recapitulate disease observed in patients since IBD is clearly multifactorial and not caused by a single mutation or defect in cellular and molecular immune pathways. As such, animal models that occur spontaneously in the absence of chemical, genetic, or immunologic manipulation are likely more representative of the human disease condition. Two animal models that spontaneously develop chronic intestinal inflammation similar to human IBD are the C3H/HeJBir and SAMP1/YitFc (SAMP) mouse strains, and their phenotypes are likely due to multiple defects in both innate and adaptive immune responses (7, 74). While gut inflammation in both models appears to be due to multiple defects, epithelial innate dysfunction also plays a central role in disease pathogenesis of these two mouse strains. As previously described, C3H/HeJBir show an impairment of epithelial innate immune responses, especially against flagellin (8, 9), leading to overaggressive adaptive immune responses. The SAMP mouse strain is another spontaneous model of IBD that most closely resembles CD for its histologic features and localization to the terminal ileum (74, 75). The ileitis characteristic of these mice is discontinuous in nature, with inflammatory lesions occurring sporadically along length of the ileum, alternating with areas of relative normalcy, and with a small percent of mice (2–3%) developing perianal fistulas (76). Alterations in epithelial morphology and architecture occur early in the disease process with expansion of IECs of primarily secretory cells lineage, including Paneth cells and goblet cells, and a decrease of mature absorptive enterocytes (77). Remarkably, *in vivo* and *ex vivo* experiments using SAMP mice have shown significantly increased epithelial paracellular permeability in the ilea in comparison to control mice; increased gut permeability was observed in both older mice with established inflammation, and in young mice (3 weeks of age), before the onset of the disease (78). This epithelial barrier defect appears to be independent of commensal bacterial colonization as increased permeability is also observed in SAMP mice raised under germ-free conditions. The increase in gut permeability is likely related to altered TJ protein expression and localization in that epithelial expression of claudin-2 is eightfold greater than in controls, while occludin is markedly suppressed, prior to the onset of intestinal inflammation. As such, the balance between different TJ proteins appears to play a crucial role in regulating TJs assembly/stabilization, and therefore, paracellular permeability. High expression of occludin and of most claudin isoforms usually reinforces the intestinal barrier; on the contrary, claudin-2 expression appears to establish lower affinity interactions with other claudin isoforms on neighboring IEC, leading to a leakier epithelial layer (79). The study of the SAMP genome revealed several quantitative trait loci with linkage to ileal inflammation, located on chromosomes 6, 8, 9, 17, and X (80). Within these loci, several potential candidate susceptibility genes have been identified, and most are involved in either immune regulation and/or intestinal epithelial functions. Among these, several were related to the structural formation of the apical junctional complex, including E-cadherin (*Cdh1*) on chromosome 8, JAM-C (*Jam3*), cingulin-like 1 (*Cgnl1*), nectin-1 (*Pvrl1*) and β-catenin (*Ctnnb1*) on chromosome 9, afadin (Mllt4) on chromosome 17, and interestingly, claudin-2 (*Cldn2*) on chromosome X (81). In addition to these data, further observations suggest a primary involvement of IEC in the pathogenesis of the ileitis characterizing SAMP mice; in fact, the primary defect appears to originate from a non-hematopoietic source since bone marrow (BM) chimeras consisting of irradiated non-inflamed control AKR recipients receiving donor pathogenic SAMP BM did not confer disease, while recipient SAMP mice receiving donor AKR BM resulted in severe ileitis (78). Taken together, these data suggest that epithelial barrier dysfunction is likely the primary, initiating trigger that leads to gut inflammation in the SAMP model of CD-like ileitis as well as other experimental models of chronic intestinal inflammation, and that is phenomenon may share similarities to patients with IBD.

It should be pointed out, however, that SAMP mice (similar to the C3H/HeJBir strain), demonstrate defects in more than one component of normal mucosal immune function. In fact, mice generated by crossing the *RAG-2* KO mutation onto the SAMP background, resulting in SAMP mice that lack mature T and B lymphocytes, do not develop ileitis (unpublished results), indicating that despite the presence of the epithelial barrier defect, the adaptive arm of the immune system is still required for the disease phenotype to occur. Therefore, the concept of “multiple hits” or defects in interacting components of host mucosal immune responses (i.e., of both innate and adaptive origin) is likely the cause of chronic intestinal inflammation in the spontaneous murine models of IBD, and is likely also necessary for the initiation and perpetuation of disease observed in patients with IBD.

### TJ protein impairment in human IBD

In IBD patients, several lines of evidence suggest that gut permeability changes could play a pivotal role in disease development; in fact, a decrease in intestinal epithelial barrier function and altered expression of TJ proteins have been observed in patients affected by CD and UC and their relatives (82, 83). Moreover, it has also been shown that in CD patients, an increase in epithelial permeability precedes episodes of disease relapse and the onset of symptoms by up to 1 year (84, 85). Interestingly, as observed in SAMP mice, claudin-2, and occludin appear to be involved in these permeability changes. IEC from IBD patients express much more claudin-2 and less occludin, claudin-3 and -4 in comparison with gut epithelia from healthy controls, particularly in active UC patients (86, 87). These findings are further corroborated by the observation that claudin-2 is markedly upregulated in the epithelium of dogs affected by idiopathic lymphocytic-plasmacytic colitis, another model of spontaneous intestinal inflammation (88).

In addition to expression data, genetic studies have also revealed a potential link between genetic polymorphisms/mutations in TJ-associated genes and the development of IBD. A recent large GWA study, including more than 2300 cases and 5400 control subjects, identified novel epithelial-related susceptibility genes for UC (89); this study found the greatest association with *HNF4A*, a gene encoding hepatocyte nuclear factor 4α, a transcription factor that regulates the synthesis of several TJ, AJ, and desmosome proteins (90). In fact, IEC-targeted deletion of *HNF4A* causes the perinatal death of experimental mice, due to severe defects in embryonic development of the gastrointestinal tract, characterized by the absence of crypt formation, reduced epithelial cell proliferation, and defective goblet cell maturation (91). In addition, conditional deletion of IEC *HNF4A* showed increased intestinal permeability and greater susceptibility to chemically induced colonic injury, suggesting the importance of this gene in intestinal inflammation (92). In the same GWA study, authors identified laminin β1 subunit (*LAMB1*) and E-cadherin (*CDH1*) as possible susceptibility genes for UC (89), corroborating the data on the dominant negative N-cadherin (62) and conditional IEC p120-catenin KO mouse models (63).

**Figure 3:**
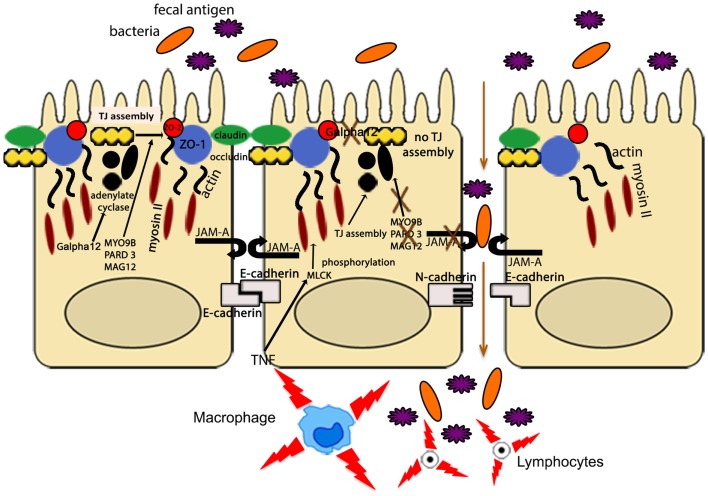
**The intestinal epithelial barrier plays a central role in gut homeostasis**. IECs form an semi-permeable lining, with barrier function modulated by the presence of TJs, AJs, and desmosomes. Expression and assembly of these protein complexes are finely regulated by several intracellular pathways. Polymorphisms in MYO9B, PARD3, and MAGI2 and impairment of the Gαi2/adenylate cyclase axis result in defective TJ assembly; phosphorylation of myosin II through MLCK activation by TNF leads to TJ disassembly. Lack of junctional proteins, such as JAM-A, or altered expression and/or pairing (e.g., dominant negative N-cadherin, claudin-2 overexpression) leads to increased epithelial permeability, facilitating translocation of luminal bacteria, and antigens and exposure to the mucosal adaptive immune system.

Other IBD susceptibility genes identified thus far appear to be primarily involved in TJ assembly. In fact, variants of myosin IXB (*MYO9B*), partitioning defective protein 3 (*PARD3*) gene, and membrane-associated guanylate kinase, WW, and PDZ domain-containing protein 2 gene (*MAGI2*), were found to be associated with UC, with a weaker association with CD for *MYO9B* and *MAGI2* (93–95). *MYO9B* encodes an unconventional myosin molecule involved in actin remodeling of epithelial enterocytes, and in TJ assembly (96), while *PARD3* and *MAGI2* encode for adaptor proteins also participating to this process (95). Interestingly, CD patients, carrying *MAGI2* variants associated with IBD, display higher serum levels of antibodies against antigens from intestinal microorganisms, such as anti-*Saccharomyces cerevisiae* (ASCA), anti-CBir1 flagellin (CBir1), and anti-outer membrane porin C (OmpC) (94), further confirming the central role of intestinal barrier function in the pathogenesis of IBD. It is worth noting that *MYO9B*, *PARD3*, and *MAGI2* were also reported to be celiac disease susceptibility genes (95, 97). If that is the case, a common, primary causal mechanism involving epithelial permeability defects may be hypothesized as a potential trigger for the development of chronic intestinal inflammation, as recapitulated in Figure 3. Again, it would be fair to say that in human disease, as postulated by the “multiple hit” theory, impairment of intestinal permeability may represent only one of the aberrancies that, if combined with others, can lead to the development of chronic intestinal inflammation, such as that observed in IBD.

### Epithelial cell integrity

**Figure 4:**
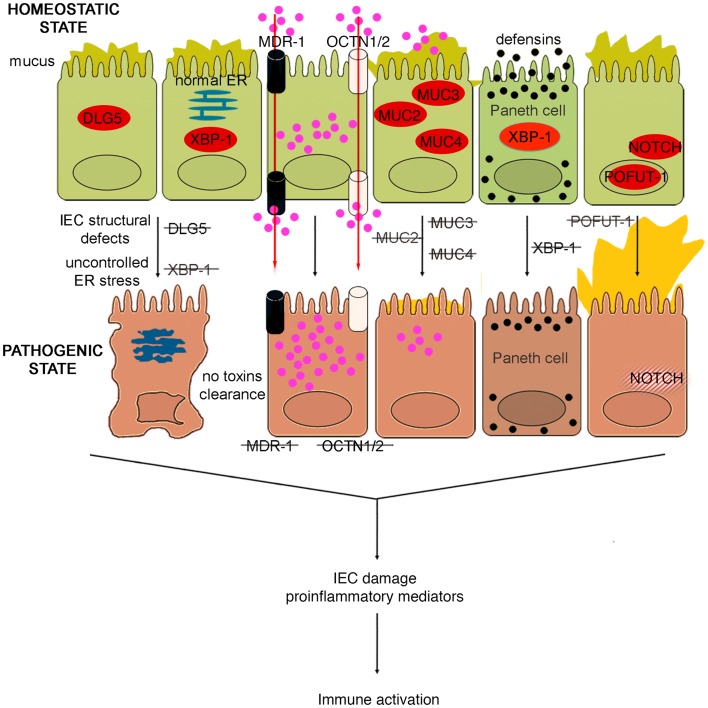
**Role of epithelial cell integrity and mucus production in gut health and disease**. Proteins regulating cell structure (e.g., DLG5) or metabolic functions (e.g., XBP1) maintain IEC integrity. IECs in constant contact with luminal toxins and xenobiotics dispose of these harmful molecules by means of several transporter proteins, such as MDR1, OCTN1, and 2. IECs secrete a thick layer of mucus, whose production is finely regulated by different proteins, including MUC family members and POFUT1. Loss of control over ER stress, resulting from XBP1 dysfunction and accumulation of toxic molecules inside IECs, secondary to transporter molecule loss of function, cause IEC damage, defective defensin secretion from Paneth cells, and release of proinflammatory mediators leading to immune activation. Direct exposure of IEC to luminal toxins/antigens is increased by deletion of MUC2, 3, and 4, which leads to dramatic reduction of mucus production, and eventually to intestinal inflammation. Conversely, overproduction of mucus is also harmful, leading to bacterial overgrowth in intestinal crypts, as seen in POFUT1 deficiency, causing a dysregulation of the epithelial transcription factor, NOTCH that controls IEC proliferation and differentiation.

The integrity and function of IECs appear to be additional determinants in intestinal barrier function. As such, alterations in structural proteins or in proteins that are pivotal in maintaining cell homeostasis may lead to a breakdown of the epithelial “wall,” as summarized in Figure 4. The profound dysfunction in IECs caused by the loss of cell polarity has the ability to trigger potent inflammatory responses within the gut. In fact, the onset of spontaneous chronic colitis has been described in mice deficient in *Ap1m2*, a master regulator of IEC polarization; these mice display impaired epithelial innate immune functions, as a consequence of a reduction in β-defensin release, followed by a pathogenic Th17 immune response (98). Inflammation also develops when normal IEC turnover is disrupted, as in the case of recombination signal protein for Ig κ J region (RPB-J); this protein is involved in the regulation of the Notch signaling pathway, which plays a major role in the regulation of intestinal epithelium differentiation and proliferation. The conditional KO of RBP-J in IECs results in the development of a spontaneous Th17 dominant colitis, characterized by impaired epithelial defense against bacteria, goblet cell hyperplasia, retarded IEC turnover, and altered TJ assembly (99). In addition, the onset of intestinal inflammation can by initiated by targeting the endoplasmic reticulum (ER) stress response, which is pivotal for the development and survival of secretory cells. Mice deficient in the transcription factor, X-box-binding protein 1 (*XBP1*), a key component in the activation of the ER stress response, spontaneously develop small intestine inflammation, which displays a patchy pattern, is not granulomatous, and has severity varying from the presence of mild polymorphonuclear infiltrates in lamina propria to the presence of crypt abscesses and ulcerations (100). Striking features of these mice are the complete ablation of functional Paneth cells, a marked reduction in number and size of small intestine goblet cells, and villus shortening with a reduced villus:crypt ratio, which are a sign of the regenerative response. In the absence of XPB1, Paneth cells are unable to process and secrete the anti-bacterial peptides and undergo early apoptosis, while small intestinal, but not colonic, goblet cells present with a reduced number of secretory granules and low levels of *MUC2* expression (100). Notably, these mice do not exhibit alteration in intestinal permeability, but are much more susceptible to *Listeria monocytogenes* infection compared to WT littermates, showing a 10-fold higher burden of *L. monocytogenes* translocating into liver and spleen 72 h after oral infection (100). Therefore, in *XBP1* KO mice, the impairment of Paneth cell function, and the consequent defect in bacterial clearance, appears to be the prominent trigger for intestinal inflammation, rather than epithelial leakiness due to suffering IECs. However, the lack of inflammation and crypt colonization by intestinal microbes in Paneth cell or cryptidin deficient mice (101) suggests that other defects are required in order to initiate intestinal inflammation. In fact, silencing of *XBP1* in the murine IEC line, MODE-K, leads to the activation of Jun N-terminal kinase (JNK)/Stress-activated protein kinase (SAPK) signaling, enhancing IEC inflammatory responses to proinflammatory stimulation, such as flagellin or TNF (100). Thus, ER stress due to knocking down *XPB1* directly puts IECs into a proinflammatory state. Even though the *XPB1* KO phenotype consists of ileitis, colonic IECs are also prone to ER stress and are, in fact, more sensitive to harmful events. As such, these mice are much more susceptible to DSS colitis than WT controls, exhibiting more severe clinical and histological signs of disease activity after the DSS challenge (100). Finally, XPB1 may also play a major role in human IBD as inflamed and non-inflamed tissue biopsies from CD and UC patients show an increased expression of this transcription factor (100). In addition, several SNPs in *XPB1* have been shown to be associated with both CD and UC (100), strongly indicating the involvement of XPB1 in human IBD pathogenesis.

The murine multiple drug resistance 1 a (*mdr1a*) gene, corresponding to human *MDR1*, encodes *P*-glycoprotein 170, which is an efflux pump for amphipathic and hydrophobic molecules, mainly xenobiotics, and is expressed in many cell lineages, including IECs (102). This molecule participates in the transmembrane transport of macromolecules, thus regulating epithelial transcellular permeability, but also in cellular detoxification processes. The importance of *P*-glycoprotein 170 in preserving intestinal homeostasis is depicted in *mdr1a* KO mice, wherein the lack of this protein causes development of spontaneous and severe colonic inflammation. The colitis phenotype, which closely resembles human UC, is characterized by massive thickening of the mucosa, leukocyte infiltration in the lamina propria, occasional crypt abscesses and ulcerations, crypt elongation, and dysregulated IEC growth (103). Not surprisingly, these mice display signs of intestinal barrier dysfunction. In fact, increased bacterial translocation that correlates to disease severity, greater basal colonic ion transport, decreased TER, are typical features of these mice. Indeed, the increase in epithelial permeability does not appear to be a consequence of the inflammation since it is observed as early as 4 weeks of age, prior to the onset of colitis (104). Moreover, the development of the disease requires the presence of the normal gut flora, as antibiotic treatment virtually prevents intestinal inflammation (103). Indeed, the genetic background greatly influences the development of the inflammatory phenotype; in fact, whereas *mdr1a* deficiency in FVB mice causes spontaneous colitis, the same genetic defect triggers colonic inflammation only in C57/BL6 mice when they are exposed to DSS (105). Gender also plays an important role in this model, as male *mdr1a* deficient mice develop severe colitis earlier and show increased epithelial permeability compared to females. In addition, barrier dysfunction is accompanied by decreased phosphorylation of the TJ proteins, occluding, and ZO-1. Interestingly, a recent study showed that colons from young, 4- to 5-week-old *mdr1a* deficient mice are disease-free and display no evidence of increased permeability compared to controls (106). In these mice, a distinct pattern of upregulated genes was observed in local tissues that are associated with bacterial recognition and the ubiquitin-proteasome system, suggesting that *P*-glycoprotein may be critical in regulating interactions with the enteric microflora leading to colitis, albeit prior to epithelial barrier disruption. Similarly to SAMP mice, the generation of BM chimeras in this model also strongly indicates an inherent epithelial defect as a primary mechanism for the development of colitis. In fact, irradiated non-inflamed control FVB recipients receiving donor pathogenic BM from *mdr1a* KO mice BM did not show signs of disease, while recipient *mdr1a* KO mice receiving donor BM from FVB mice developed overt colitis. Taken together, these data suggest that the initiating factor for the development of colitis in *mdr1a* KO mice is likely the result of an epithelial-derived dysfunction; however, controversy remains as to whether the primary event is solely due to a permeability defect, given the expression of *P*-glycoprotein 170 in hematopoietic cells as well.

In support of the relevance of this transporter in gut inflammation, a polymorphism (Ala893) of *MDR1* has been reported to be associated to IBD (107). Likewise, IBD genetic studies identified two genes, encoding epithelial transporter proteins, as IBD susceptibility genes. Solute carrier 22A4/organic cation transporter 1 (*SLC22A4/OCTN1*) and *SLC22A5/OCTN2*, localized on chromosome 5, encode transporter proteins involved in xenobiotic and toxin removal, physiological substrate uptake, and carnitine metabolism (108). Functional nucleotide polymorphisms (SNPs) of these genes, leading to impairment of OCTN promoter activity and consequently to severe alterations in transporter functions, were found to confer susceptibility to the development of CD (109). Therefore, it could be hypothesized that the carriage of *MDR1*, *OCTN1*, and *OCTN2* variants might interfere with IEC homeostasis at different levels: variants of all three of these genes could alter transepithelial permeability to large molecules, carriage of the *MDR1* polymorphism could dampen the ability of IEC to eliminate potentially toxic molecules, while *OCTN1* and *OCTN2* SNPs could damage mucosal energetic metabolism through a defective carnitine intake.

Other genetic variants that may affect the integrity of IEC structure and polarity could represent alternative predisposing factors for the onset of IBD. In fact, variants of the gene encoding the structural protein Disk large homolog 5 (*DLG5*) appear to predispose individuals to CD (110). *DLG5* is located on chromosome 10 and encodes a scaffolding protein expressed in IEC from both the colon and small bowel, and is involved in the maintenance of IEC integrity, regulation of cell growth, as well as epithelial polarization (111). Haplotype D, or R30Q variant (SNP 113G → A), influences CD susceptibility as it encodes an amino acid substitution that results in a mutation that likely interferes with DLG5 scaffolding (110). Similar to *MYOIXB*, *PARD3*, and *MAGI2* variants, the *DLG5 R30Q* polymorphism has also been reported to be associated with celiac disease (112), corroborating the concept that a common mechanism, that is breakdown of the intestinal barrier, may exist between celiac disease and IBD. In addition to the scaffolding function, which also involves the constitution of AJs, DLG5 is also important in innate immune responses. Recent studies have identified an exon, which encodes for a CARD domain, that has been identified within the *DLG5* gene and its expression in colonic tissues has been confirmed (113). As such, *DLG5* may belong, or be closely related to, the *CARD/NOD* family, and therefore, may also participate directly in bacteria-host interactions within the gut. Interestingly, data generated in pediatric and adult IBD populations showed a gender effect in analyzing *DLG5 R30Q* carriage and CD susceptibility. While this haplotype represents a susceptibility factor for CD in males, it confers protection against the development of disease in females (114, 115). Further studies, however, are warranted to investigate whether gender differences exist and to mechanistically determine how *DLG5* is involved in IBD pathogenesis.

### Genetic regulation of epithelial mucus production

Aside from their absorptive, immunological, and barrier functions, IECs are also specialized to produce a large amount of mucus. As such, the epithelial mucosal surface is covered by a more than 100 μm thick layer of mucus, secreted by goblet cells (116). The purpose of this mucus layer, aside from the lubrication between the luminal contents of the gut and the epithelial surface, is to add further protection to the intestinal barrier. The main constituents of mucus are phospholipids and mucins, both of which are highly negatively charged; therefore, the mucus layer represents both a mechanical and chemical barricade overlying the IEC lining. In addition, the mucus layer also supports the presence of proteins in close proximity to the intestinal wall that are pivotal in controlling luminal bacterial burden, such as secretory IgA (117) and lactoferrin (118).

Goblet cells may also play a primary role in the activation of the intestinal inflammatory process. Goblet cells contain potent regulators of the inflammatory cascade, such as components of the kallikrein-kinin system, within their cytoplasmic secretory granules. Kallikrein is an enzyme present in different isoforms, in plasma, and in tissues that cleaves high molecular weight kininogen to release bradykinin and activates both coagulation and inflammatory events (119). Conversely, kallistatin, a member of the serine proteinase inhibitor family, is its specific antagonist (120). Tissue expression of kallikrein and kallistatin significantly varies in active IBD; in fact, localization in normal intestinal tissues, in non-involved area of IBD patients, and in specimens from diverticulitis patients, is confined to the cytoplasm of intestinal goblet cells. During the specific inflammatory process characterizing active IBD, goblet cells are depleted of both kallikrein and kallistatin, which are, instead, massively present in the interstitium (121, 122). Thus, goblet cells appear to actively secrete kallikrein and kallistatin in the interstitium, directly regulating local gut inflammatory responses.

Similar to that observed with other components that are important in maintaining intestinal barrier function, dysfunction of mucus production may also lead to intestinal inflammation. For example, while *MUC2* KO mice lack the complete gene for MUC2 mucin (123), different strains of *MUC2* mutant mice, Winnie and Eeyore, are characterized by two distinct non-complementing missense mutations in *MUC2* (124). The different mouse strains spontaneously develop colitis, exhibiting watery diarrhea, rectal bleeding, and prolapse. Histological features include mucosal thickening, superficial erosions, crypt elongation, goblet cell loss, neutrophilic infiltration, and crypt abscesses. Surprisingly, the colitic phenotype of Winnie and Eeyore mice appears to be worse and more penetrant than *MUC2* KO mice, the latter being present only under certain genetic backgrounds (124). This phenomenon could be explained considering that the mutations in Winnie and Eeyore mice cause MUC2 protein misfolding and a consequential accumulation in the cytoplasm leading to ER stress (124). This observation, again, highlights the importance of the “multiple hit” concept, since the complete lack of *MUC2*, predisposing to bowel inflammation, is not sufficient to initiate the cascade of molecular events leading to intestinal inflammation. If the impairment of the mucus barrier is associated with other pathogenic noxae, for example, the disease manifests itself in full.

In fact, reduced production of MUC2 has been reported in human IBD, particularly UC, although it is not well understood whether this decrease represents a primary defect or if it is secondary to the epithelial damage induced by inflammation (125). However, genetic polymorphisms involved in the regulation of mucus production have also been associated with human IBD. In particular, it has been suggested that a few, rare variable number of tandem repeat (VNTR) alleles of the human intestinal mucin gene, *MUC3*, and non-synonymous SNPs of *MUC3A*, which is part of the *MUC3* gene and encodes a membrane-bound mucin with epidermal growth factor (EGF)-like motifs that alter IEC signaling, may confer a genetic predisposition to UC (126) and CD (127), respectively. Thus, impairment of the bowel mucosal barrier, due to different *MUC3A* variants, may be involved in the pathogenesis of both UC and CD. On the other hand, an hyperproduction of mucus, leading to an alteration of mucus-associated flora has been implicated as the basis for the enterocolitic phenotype presented by mice with selective deletion of protein *O*-fucosyltransferase 1 (*Pofut1*) in IECs (128). POFUT1 is an enzyme required for correct signaling of the Notch pathway. Mice defective in IEC-specific POFUT1 display marked hyperplasia and hypertrophy of goblet cells in both the small intestine and colon, leading to an oversecretion of mucus. Other features of these mice are Paneth and enteroendocrine cell hyperplasia (128). At 4 weeks of age, these mice start to develop ileal and colonic inflammation, evident by thickening of the intestinal wall and by an increase in intestinal permeability. Inflammation increases until 36 weeks of age, with 100% penetrance at this age. The enterocolitis is histologically characterized by crypt hyperplasia, inflammatory infiltrates within the lamina propria, crypt abscesses, and transmural inflammation. Moreover, significantly high levels of Th1 and Th17 cytokines are detectable in the inflamed tissues, while a shift toward Gram-negative bacteria is evident in the gut microflora, with spiral-shaped organisms accumulating in dilated and mucus filled crypts (128).

In further support of intestinal mucus production playing a pivotal role in maintaining gut homeostasis, is the observation that thickening of mucus secretions, secondary to the common cystic fibrosis transmembrane conductance regulator (*CFTR*) ΔF508 mutation, appears to confer protection against the development of CD. In fact, heterozygosity for this mutation has recently been shown to be negatively associated with CD in two independent European cohorts, but had no impact on the risk of developing UC (129). *CFTR* encodes a transmembrane transporter that pumps chloride anions out of the cell, regulating both secretory and absorptive functions, and the production of mucus from IECs; however, it can also bind the TLR4 ligand, LPS, impacting on the interactions between IECs and bacteria. The ΔF508 mutation causes loss of one phenylalanine from the CFTR amino acid sequence and consequently, the misfolding of this protein. This mutation completely eradicates the functional activity of CFTR, and therefore TER is increased, while PRR signaling remains intact (129). The fact that carriage of one allele of this mutation confers protection against CD further underscores the importance of genetic contributions in regulating epithelial barrier function, as well as intestinal permeability and epithelial innate responses, in maintaining normal mucosal immune homeostasis.

## Therapies That Enhance Intestinal Barrier Function in IBD

**Figure 5:**
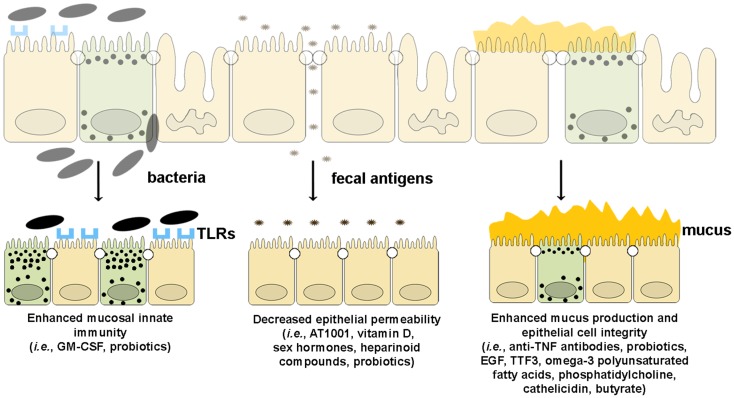
**Therapeutic agents that enhance epithelial barrier function**. Several drugs can potentially improve different components of intestinal barrier function by (from left to right): (1) enhancing mucosal innate immunity through increased expression of TLRs and production of anti-microbial peptides, (2) decreasing epithelial permeability through the expression and assembly of TJ and AJ proteins, and (3) restoring epithelial cell and mucus layer integrity by reducing IEC apoptosis and inducing mucus production.

Since emerging evidence in recent years has implicated the importance of intestinal barrier function in maintaining gut immune homeostasis, increasing efforts and investment in developing tools by which to manipulate epithelial innate immunity and permeability have been made in order to obtain therapeutic effects in human disease. At present, several approaches have been made and/or proposed to enhance intestinal barrier function using several different strategies. Such drug development could boost epithelial innate immunity, decrease the permeability of the epithelial barrier, or improve the quality and quantity of mucus production (Figure 5).

### Enhancers of epithelial innate immunity

The use of granulocyte-monocyte colony stimulating factor (GM-CSF), or Sargramostim, has been proposed for the treatment of patients suffering from CD. A randomized placebo controlled clinical trial demonstrated a significant improvement in disease activity in patients treated with GM-CSF compared to those treated with placebo (130), even though the clinical remission rates did not differ in the two groups. As such, instead of serving as an immunosuppressant or anti-inflammatory agent, GM-CSF has been proposed to serve an enhancer of innate immune responses and has been used for the treatment of inflammatory diseases. This paradox may be explained by considering that GM-CSF is capable of boosting innate immune functions, and therefore, may reinforce the intestinal mucosal barrier (131). In fact, GM-CSF stimulation increases neutrophil expression of PRRs (132), and neutrophil, monocyte, and macrophage bactericidal activity (133). At present, most of the data regarding GM-CSF’s effects on innate immunity come from experiments involving hematopoietic cells since it has been shown that IECs, including Paneth cells, display receptors for GM-CSF, and proliferate after GM-CSF stimulation (134). It is therefore plausible that GM-CSF may have a direct influence on IEC barrier function.

Several lines of evidence also suggest that probiotics can modulate host epithelial innate immune responses. Indeed, it is believed that probiotics have the ability to stimulate mucosal defenses through TLRs and upregulation of innate-type cytokines (135, 136). For example, experimental data has shown that administration of *Lactobacillus casei* has the ability to potently increase cellular expression of TLR2 (137), and the *E. coli* strain, Nissle 1917, can induce the expression of β-defensins (138), as well as both TLR2 and TLR4 (139). Moreover, *E. coli* strain Nissle 1917 has been shown to ameliorate DSS colitis in C57BL/6 WT mice, but not in mice deficient in *TLR2* or *TLR4*, suggesting that activation of both TLR2 and TLR4 signaling is pivotal for this microorganism in order to exert its beneficial effects (139). Enhancement of innate immune function is not the only way by which probiotics may aid in the control of intestinal pathogens; probiotic supplementation can alter the microflora of IBD patients (140) through competition with enterotoxigenic and enteropathogenic bacteria for energy sources and for IEC surface receptors. In this way, probiotics may block binding of pathogens to the intestinal epithelial surface, inhibiting their invasivity, and thereby reducing potential bacterial translocation (141, 142).

### Drugs that decrease epithelial permeability and leakiness

The modulation of TJ protein expression may also represent a promising target for novel therapies for the treatment of inflammatory gut disorders. At present, the use of compounds with the ability to enhance TJ expression and function has already been proposed for celiac disease. Indeed, celiac disease shares several features with IBD, including an increase in intestinal permeability, and as mentioned earlier, a few susceptibility genes involved in TJ assembly. An octapeptide, called AT-1001, derived from *Vibrio cholera*’s zonula occludens toxin (ZOT), has been proposed for the treatment of celiac disease. This peptide has the ability to interfere with IEC cytoskeleton rearrangement and TJ disassembly, secondary to gliadin exposure (143). Thus, AT-1001 antagonizes the increase in paracellular permeability induced by ZOT analogs, and the effect of gliadin on epithelial permeability in the duodenal mucosa of celiac patients (143). In addition, IL-10 KO mice that develop spontaneous colitis displayed less severe intestinal disease when treated with AT-1001 (144). Therefore, the rationale for the use of AT-1001 in gut inflammatory disorders has become quite apparent. Currently, only two randomized placebo-controlled safety and dose-ranging studies, involving 20 and 86 celiac disease patients, have been performed, demonstrating the safety and tolerability of this molecule and its ability to reduce intestinal permeability (143, 145). Larger clinical studies are warranted in order to thoroughly evaluate the therapeutic value of this octapeptide in celiac disease, and possibly, in IBD.

Vitamin D has also been shown to have the potential of reducing epithelial permeability and ameliorating mucosal inflammation in IBD. Vitamin D induces the expression of several TJ proteins, such as ZO-1, claudin-1, claudin-2 (146), as well as the AJ protein, E-cadherin (147). Vitamin D is also able to increase the TER in monolayers of Caco-2 cells by augmentation of junction protein expression (146), demonstrating a functional effect. In addition, knockdown of the Vitamin D receptor (*VDR*) gene in Caco-2 cells causes a marked reduction in IEC TJ expression and function, while *VDR* KO mice are more susceptible to DSS-induced colitis and exhibit more severe inflammation compared to WT littermates. These mutant mice display delayed and impaired wound healing and a significant reduction in colonic TER following DSS challenge, which is related to the marked destruction of TJ complexes (146). Remarkably, carriage of *VDR* genetic polymorphisms has been associated with the development of human IBD in different populations (148, 149). Thus, Vitamin D appears to play a critical role in maintaining intestinal barrier function, although further data are needed to rationalize its clinical use in patients with IBD.

Sex hormones, as well, may play an important role in the modulation of immune responses and intestinal permeability. It is well established that the prevalence of inflammatory and autoimmune disorders is higher in female than in male subjects (150), and this gender bias is present, albeit to a much lesser extent, in IBD patients. In fact, CD is slightly more common and aggressive in female compare to male patients (151). However, how estrogens and androgens can modify mucosal immune responses still remains somewhat obscure. The administration of dehydroepiandrosterone and 7-alpha-hydroxy-dehydroepiandrosterone in rats with TNBS colitis attenuated the degree of mucosal damage and inflammation (152); surprisingly, despite the potential role of estrogens in triggering autoimmunity, 17alpha-ethynyl-17beta-estradiol also ameliorated the severity of disease in HLA-B27 transgenic rats, which spontaneously develop colitis (153). One possible explanation is differential signaling through the estrogen receptors (ER), ERα, and ER-β. Interestingly, it has been demonstrated that activation of ERβ on IECs, through estradiol or specific agonists, increases epithelial barrier function by upregulating occludin and JAM-A, both *in vitro* and *in vivo* systems (154). Thus, alterations in TJs triggered by selective ERβ activation may be protective against the development of intestinal inflammation, suggesting a possible role of ERβ agonists in the treatment of IBD.

Parenteral heparin and low molecular weight heparins (LMWHs), such as tinzaparin, deligoparin, enoxaparin, reviparin, and dalteparin, have been proposed as a therapeutic option in IBD, primarily due to their anti-inflammatory properties. Their actual efficacy, however, is debatable, at least considering the parenteral administration route (155). Data recently obtained using chemically induced colitis models suggest a potential role of two different LMWHs in ameliorating gut inflammation if administered directly on the epithelial surface. In one study, parnaparin, a LMWH of 5000 kD, was administered to rats intracolonically, and improved disease severity (156). In another study, colitic mice treated with microspheres loaded with enoxaparin (4500 kD), specifically designed to release the drug into the colon, resulted in a reduction of inflammatory activity after parenteral administration (157). The same approach was tested in a pilot study in patients with mild to moderate active distal UC; sodium parnaparin, dispersed in a multimatrix formulation (MMX) in order to obtain a controlled colonic-release of the drug, showed no side effects and possible efficacy (158). A later, randomized, placebo-controlled clinical trial validated these results, showing a significant clinical response in parnaparin- vs. placebo-treated patients (159). The potential efficacy of heparins may be explained not only by their primary anti-inflammatory properties, but also their ability to regulate epithelial permeability. In fact, the lack of heparan sulfate and syndecan-1, two proteoglycans belonging to the heparinoid compound family, from the basolateral surface of IECs is closely linked with protein-losing enteropathy (PLE), a syndrome, characterized by the leakage of proteins into the intestinal lumen through the gut wall (160–162). Knockdown of syndecan-1 causes augmented paracellular permeability in *in vitro* monolayers of the HT29 colon cancer cell line, while heparan sulfate and syndecan-1 deficient mice display increased intestinal protein loss that can be prevented by the parenteral administration of heparins, including 2,3-de-*O*-sulfated heparin, which does not have any anticoagulant properties (163).

Probiotics, as well, appear influence epithelial paracellular permeability, as indicated by the increased TER obtained following treatment of *in vitro* intestinal epithelial monolayers (164). The mechanism(s) by which intestinal permeability is decreased following probiotic treatment involves rearrangement of TJ proteins, such as ZO-2, protein kinase C ζ (PKCζ) and occludins, that are pivotal in stabilizing TJ complexes (165, 166). It has also been shown that probiotics can decrease intestinal permeability, *in vivo*, during chemically induced colitis, restoring gut barrier integrity through the maintenance of TJ protein expression, and the prevention of IEC apoptosis (167). Accordingly, VSL#3, a multiple probiotic formulation, proved to be efficacious in restoring early epithelial permeability defects in young SAMP mice, preventing/delaying the development of intestinal inflammation. This effect appears to be related to the increase of TNF production by IECs (168); in fact, co-administration of VSL#3 and anti-TNF antibodies significantly dampened the efficacy of probiotics (169). Thus, increased TNF production by IECs induced by probiotics, may enhance different components of mucosal innate immunity and/or epithelial barrier function.

### Restoration of epithelial cell integrity and biology

The integrity of IECs themselves, their cell membrane, and the mucus layer covering the epithelium of the entire gastrointestinal tract, are of paramount importance in maintaining gut immune homeostasis. Indeed, a defect in mucus production has been suggested as a primary feature of UC since the 1980s (170).

Anti-TNF monoclonal antibody therapies have shown great efficacy in obtaining and/or maintaining clinical and endoscopic remission in patients with IBD (171). Aside from their potent immunomodulating effects, anti-TNF antibody administration has the ability to restore mucosal barrier integrity, normalizing intestinal permeability, in CD patients (172, 173). According to experimental data from studies on human endoscopic biopsies (174) and on SAMP mice (175), this effect could be related to homeostatic regulation of mucosal cell apoptosis induced by anti-TNF strategies. Similarly, manipulation of the enteric flora may provide an alternative strategy to increase epithelial barrier integrity by inducing anti-apoptotic processes. Administration of specific commensal *E. coli* in a mouse model of intestinal development was shown to prevent staurosporine-induced apoptosis, increasing the tissue expression of IFNαA and guanylate binding protein-1 (GBP-1), a recently identified anti-apoptotic protein (176).

Growth factors and mediators that promote mucosal wound healing have also been proposed to enhance epithelial barrier integrity by inducing epithelial repair and restitution processes. EGF, for example, has been proposed and tested as a potential therapeutic agent for the treatment of UC. The addition of EGF enemas to mesalamine treatment in UC patients has been shown to increase the response rate to the therapy, confirming the importance of enhancing mucosal integrity for IBD patients (177). In addition, as mentioned earlier, trefoil factors are proteins synthesized by IECs that initiate and improve wound healing after a mucosal injury. Selective deletion of TFF3, abundantly produced by goblet cells in both the large and small intestine, increases the susceptibility to chemically induced colitis (178). Interestingly, exogenous administration of TFF3 was able to reverse colitis, suggesting a potential role of TFF3 in IBD management (178). As such, a pilot clinical trial using human recombinant TFF3 enemas as a novel therapeutic strategy for the treatment of UC was performed (179); this trial, however, did not show any significant difference between patients treated with topical TFF3 in combination with oral 5-ASA vs. those treated with oral 5-ASA alone. Therefore, further studies, perhaps with different clinical conditions or with different administration routes are needed in order to investigate the true therapeutic value of this molecule.

The intestinal barrier can also be structurally manipulated through modification of the IEC membrane composition. Administration of sphingomyelinase decreases TER in monolayers of Caco-2 cells (180) through the hydrolysis of IEC membrane sphingomyelin into ceramide (181), thus altering the composition of cholesterol and sphingolipids in TJ membrane microdomains (182) and transmembrane signaling (180). The end result of this complex chain of molecular events leads, in the end, to an increase in paracellular and transcellular epithelial permeability. Similarly, intestinal epithelial permeability is directly modified by cell membrane lipid content (183–185). As such, omega-3 polyunsaturated fatty acids have been proposed as an adjuvant therapy in IBD (186, 187), even though their efficacy in inducing and maintaining remission is still debatable (188, 189). Interestingly, recent randomized and controlled clinical trials showed that the administration of phosphatidylcholine by slow release ameliorates the inflammatory activity in UC patients, also in steroid refractory subset of patients (190, 191). The rationale behind this rather new therapeutic approach is that phosphatidylcholine and other phospholipids (192) serve as major components of the intestinal mucus layer, generating a hydrophobic protective layer overlying IECs, and therefore take part in establishing the gut mucosal barrier. Mucus from UC patients has been shown to be defective in phosphatidylcholine compared to controls and CD patients (192); thus, this deficiency could be of pivotal importance in the pathogenesis of disease since it significantly alters colonic barrier functions. In addition, intestinal mucus production can be boosted by the administration of cathelicidin, an anti-microbial peptide secreted by IECs. Cathelicidin has been given intrarectally to mice challenged with DSS; in this experiment, mice treated with cathelicidin displayed increased mucus production, an overexpression of the mucin genes *MUC1*, *MUC2*, *MUC3*, and *MUC4*, and milder colitis than untreated controls (193). The ability of cathelicidin to induce mucin gene expression has been confirmed, in part, in the colonic epithelial cell line, HT-29, which responded by upregulating *MUC1* and *MUC2*, but not *MUC3* and *MUC4* (193). Therefore, aside from its bactericidal activity, cathelicidin may have the potential to treat colitis due to its effect on enhancing gut mucus production.

Similar to cathelicidin, butyrate, a short chain fatty acid produced by intestinal microbial fermentation of dietary fibers, has the ability to reinforce epithelial barrier function through an increase in mucus production. In fact, administration of butyrate to human colon cancer cell lines or to endoscopic colon biopsies clearly upregulates mucin production (194, 195), while intrarectal delivery of butyrate into rats increased colonic mucus secretion (196). As such, several studies suggest a primary role of butyrate in the modulation of epithelial permeability. Butyrate can increase TER in Caco-2 and HT-29 cell monolayers by augmenting TJ protein expression (197, 198); however, this effect appears to be dose and cell type dependent. In fact, a higher concentration of butyrate increased paracellular permeability in Caco-2 monolayers (198) and in rat distal colon specimens (199). Interestingly, butyrate exerts many other effects on IECs, specifically contributing to the energetic balance of cells, controlling oxidative stress, and in regulating the inflammatory status of cells (200). Its role in maintaining the epithelial barrier may be more complex, including several pivotal functions for the preservation of IEC homeostasis and integrity. Currently, it is unclear which of these many actions confers the most potent therapeutic effect induced by butyrate administration (201, 202).

In summary, several therapeutic agents demonstrate the potential of modifying the intestinal barrier and enhancing various IEC functions. Their true clinical efficacy in the management of chronic intestinal inflammatory disorders, however, is still largely unknown. This is primarily due to the lack or scarcity of evidence-based information and data regarding the effectiveness of these compounds for the treatment of intestinal inflammation. Therefore, while a deeper knowledge of the cellular events leading to the impairment of mucosal barrier function and the onset of gut inflammation is needed, more safety and efficacy trials are warranted to assess the feasibility of manipulating the intestinal barrier as a novel therapeutic approach for the treatment of chronic inflammation in the GI tract.

## Conclusion

The etiology of IBD is complex and as researchers deepen their knowledge regarding the mechanisms underlying the pathogenesis of chronic intestinal inflammation, emerging evidence has revealed that the intestinal epithelium plays a central role. Defects of primary epithelial etiology leading to chronic gut inflammation globally include dysfunction of innate immune responses and of epithelial barrier integrity. However, it is likely that the development of IBD occurs as the result of a concomitant presence of different defects in various compartments, as postulated by the “multiple hit” theory, and as supported by several mouse model of chronic intestinal inflammation. As such, impairment of normal intestinal epithelial function, although likely not sufficient by itself to sustain the inflammatory process, plays a primary role in the onset and maintenance of disease. Further investigation is needed to define its precise role in the pathogenesis of IBD, which will lead to more targeted therapies and strategic approaches to specifically boost intestinal epithelial function to ultimately treat patients with IBD.

## Conflict of Interest Statement

The authors declare that the research was conducted in the absence of any commercial or financial relationships that could be construed as a potential conflict of interest.

## References

[1] Medzhitov R Janeway C Jr Innate immunity. N Engl J Med (2000) 343:338–44 10.1056/NEJM200008033430506 10922424

[2] Akira S Takeda K Toll-like receptor signalling. Nat Rev Immunol (2004) 4:499–511 10.1038/nri1391 15229469

[3] Gay NJ Keith FJ *Drosophila* toll IL-1 receptor. Nature (1991) 351:355–6 10.1038/351355b0 1851964

[4] Belvin MP Anderson KV A conserved signaling pathway: the *Drosophila* toll-dorsal pathway. Annu Rev Cell Dev Biol (1996) 12:393–416 10.1146/annurev.cellbio.12.1.393 8970732

[5] Vijay-Kumar M Sanders CJ Taylor RT Kumar A Aitken JD Sitaraman SV Deletion of TLR5 results in spontaneous colitis in mice. J Clin Invest (2007) 117:3909–21 18008007 10.1172/JCI33084 PMC2075480

[6] Carvalho FA Nalbantoglu I Ortega-Fernandez S Aitken JD Su Y Koren O Interleukin-1beta (IL-1beta) promotes susceptibility of toll-like receptor 5 (TLR5) deficient mice to colitis. Gut (2012) 61:373–84 10.1136/gut.2011.240556 21646247

[7] Sundberg JP Elson CO Bedigian H Birkenmeier EH Spontaneous, heritable colitis in a new substrain of C3H/HeJ mice. Gastroenterology (1994) 107:1726–35 7958684 10.1016/0016-5085(94)90813-3

[8] Beckwith J Cong Y Sundberg JP Elson CO Leiter EH Cdcs1, a major colitogenic locus in mice, regulates innate and adaptive immune response to enteric bacterial antigens. Gastroenterology (2005) 129:1473–84 10.1053/j.gastro.2005.07.057 16285949

[9] Lodes MJ Cong Y Elson CO Mohamath R Landers CJ Targan SR Bacterial flagellin is a dominant antigen in Crohn disease. J Clin Invest (2004) 113:1296–306 15124021 10.1172/JCI20295 PMC398429

[10] Farmer MA Sundberg JP Bristol IJ Churchill GA Li R Elson CO A major quantitative trait locus on chromosome 3 controls colitis severity in IL-10-deficient mice. Proc Natl Acad Sci U S A (2001) 98:13820–5 10.1073/pnas.241258698 11707574 PMC61125

[11] Carvalho FA Barnich N Sauvanet P Darcha C Gelot A Darfeuille-Michaud A Crohn’s disease-associated *Escherichia coli* LF82 aggravates colitis in injured mouse colon via signaling by flagellin. Inflamm Bowel Dis (2008) 14:1051–60 10.1002/ibd.20423 18338780

[12] Boudeau J Glasser AL Masseret E Joly B Darfeuille-Michaud A Invasive ability of an *Escherichia coli* strain isolated from the ileal mucosa of a patient with Crohn’s disease. Infect Immun (1999) 67:4499–509 10456892 10.1128/iai.67.9.4499-4509.1999 PMC96770

[13] Abraham C Cho JH Bugging of the intestinal mucosa. N Engl J Med (2007) 357:708–10 10.1056/NEJMcibr073420 17699823

[14] Gewirtz AT Vijay-Kumar M Brant SR Duerr RH Nicolae DL Cho JH Dominant-negative TLR5 polymorphism reduces adaptive immune response to flagellin and negatively associates with Crohn’s disease. Am J Physiol Gastrointest Liver Physiol (2006) 290:G1157–63 10.1152/ajpgi.00544.2005 16439468

[15] Fukata M Michelsen KS Eri R Thomas LS Hu B Lukasek K Toll-like receptor-4 is required for intestinal response to epithelial injury and limiting bacterial translocation in a murine model of acute colitis. Am J Physiol Gastrointest Liver Physiol (2005) 288:G1055–65 10.1152/ajpgi.00328.2004 15826931

[16] Cario E Podolsky DK Differential alteration in intestinal epithelial cell expression of toll-like receptor 3 (TLR3) and TLR4 in inflammatory bowel disease. Infect Immun (2000) 68:7010–7 10.1128/IAI.68.12.7010-7017.2000 11083826 PMC97811

[17] Franchimont D Vermeire S El Housni H Pierik M Van Steen K Gustot T Deficient host-bacteria interactions in inflammatory bowel disease? The toll-like receptor (TLR)-4 Asp299gly polymorphism is associated with Crohn’s disease and ulcerative colitis. Gut (2004) 53:987–92 10.1136/gut.2003.030205 15194649 PMC1774122

[18] Pierik M Joossens S van Steen K Van Schuerbeek N Vlietinck R Rutgeerts P Toll-like receptor-1, -2, and -6 polymorphisms influence disease extension in inflammatory bowel diseases. Inflamm Bowel Dis (2006) 12:1–8 10.1097/01.MIB.0000195389.11645.ab 16374251

[19] Cario E Gerken G Podolsky DK Toll-like receptor 2 controls mucosal inflammation by regulating epithelial barrier function. Gastroenterology (2007) 132:1359–74 10.1053/j.gastro.2007.02.056 17408640

[20] Podolsky DK Gerken G Eyking A Cario E Colitis-associated variant of TLR2 causes impaired mucosal repair because of TFF3 deficiency. Gastroenterology (2009) 137:209–20 10.1053/j.gastro.2009.03.007 19303021 PMC2812790

[21] Pedersen G Andresen L Matthiessen MW Rask-Madsen J Brynskov J Expression of toll-like receptor 9 and response to bacterial CpG oligodeoxynucleotides in human intestinal epithelium. Clin Exp Immunol (2005) 141:298–306 10.1111/j.1365-2249.2005.02848.x 15996194 PMC1809430

[22] Torok HP Glas J Tonenchi L Bruennler G Folwaczny M Folwaczny C Crohn’s disease is associated with a toll-like receptor-9 polymorphism. Gastroenterology (2004) 127:365–6 10.1053/j.gastro.2004.05.051 15236225

[23] Nenci A Becker C Wullaert A Gareus R van Loo G Danese S Epithelial NEMO links innate immunity to chronic intestinal inflammation. Nature (2007) 446:557–61 10.1038/nature05698 17361131

[24] Kajino-Sakamoto R Inagaki M Lippert E Akira S Robine S Matsumoto K Enterocyte-derived TAK1 signaling prevents epithelium apoptosis and the development of ileitis and colitis. J Immunol (2008) 181:1143–52 18606667 10.4049/jimmunol.181.2.1143 PMC3065656

[25] Hugot JP Chamaillard M Zouali H Lesage S Cezard JP Belaiche J Association of NOD2 leucine-rich repeat variants with susceptibility to Crohn’s disease. Nature (2001) 411:599–603 10.1038/35079107 11385576

[26] Ogura Y Bonen DK Inohara N Nicolae DL Chen FF Ramos R A frameshift mutation in NOD2 associated with susceptibility to Crohn’s disease. Nature (2001) 411:603–6 10.1038/35079114 11385577

[27] Ogura Y Inohara N Benito A Chen FF Yamaoka S Nunez G Nod2, a Nod1/Apaf-1 family member that is restricted to monocytes and activates NF-kappaB. J Biol Chem (2001) 276:4812–8 10.1074/jbc.M008072200 11087742

[28] Ogura Y Lala S Xin W Smith E Dowds TA Chen FF Expression of NOD2 in Paneth cells: a possible link to Crohn’s ileitis. Gut (2003) 52:1591–7 10.1136/gut.52.11.1591 14570728 PMC1773866

[29] Inohara N Ogura Y Fontalba A Gutierrez O Pons F Crespo J Host recognition of bacterial muramyl dipeptide mediated through NOD2. Implications for Crohn’s disease. J Biol Chem (2003) 278:5509–12 10.1074/jbc.C200673200 12514169

[30] Schreiber S Rosenstiel P Albrecht M Hampe J Krawczak M Genetics of Crohn disease, an archetypal inflammatory barrier disease. Nat Rev Genet (2005) 6:376–88 10.1038/nrg1607 15861209

[31] Abbott DW Wilkins A Asara JM Cantley LC The Crohn’s disease protein, NOD2, requires RIP2 in order to induce ubiquitinylation of a novel site on NEMO. Curr Biol (2004) 14:2217–27 10.1016/j.cub.2004.12.032 15620648

[32] Lala S Ogura Y Osborne C Hor SY Bromfield A Davies S Crohn’s disease and the NOD2 gene: a role for Paneth cells. Gastroenterology (2003) 125:47–57 10.1016/S0016-5085(03)00661-9 12851870

[33] Wehkamp J Schmid M Fellermann K Stange EF Defensin deficiency, intestinal microbes, and the clinical phenotypes of Crohn’s disease. J Leukoc Biol (2005) 77:460–5 10.1189/jlb.0904543 15618294

[34] Van Limbergen J Russell RK Nimmo ER Ho GT Arnott ID Wilson DC Genetics of the innate immune response in inflammatory bowel disease. Inflamm Bowel Dis (2007) 13:338–55 10.1002/ibd.20096 17206667

[35] Ganz T Defensins: antimicrobial peptides of innate immunity. Nat Rev Immunol (2003) 3:710–20 10.1038/nri1180 12949495

[36] Koslowski MJ Kubler I Chamaillard M Schaeffeler E Reinisch W Wang G Genetic variants of Wnt transcription factor TCF-4 (TCF7L2) putative promoter region are associated with small intestinal Crohn’s disease. PLoS One (2009) 4:e4496 10.1371/journal.pone.0004496 19221600 PMC2637978

[37] Wehkamp J Harder J Weichenthal M Schwab M Schaffeler E Schlee M NOD2 (CARD15) mutations in Crohn’s disease are associated with diminished mucosal alpha-defensin expression. Gut (2004) 53:1658–64 10.1136/gut.2003.032805 15479689 PMC1774270

[38] Voss E Wehkamp J Wehkamp K Stange EF Schroder JM Harder J NOD2/CARD15 mediates induction of the antimicrobial peptide human beta-defensin-2. J Biol Chem (2006) 281:2005–11 10.1074/jbc.M511044200 16319062

[39] Takahashi N Vanlaere I de Rycke R Cauwels A Joosten LA Lubberts E IL-17 produced by Paneth cells drives TNF-induced shock. J Exp Med (2008) 205:1755–61 10.1084/jem.20080588 18663129 PMC2525583

[40] Harrington LE Hatton RD Mangan PR Turner H Murphy TL Murphy KM Interleukin 17-producing CD4+ effector T cells develop via a lineage distinct from the T helper type 1 and 2 lineages. Nat Immunol (2005) 6:1123–32 10.1038/ni1254 16200070

[41] Langrish CL Chen Y Blumenschein WM Mattson J Basham B Sedgwick JD IL-23 drives a pathogenic T cell population that induces autoimmune inflammation. J Exp Med (2005) 201:233–40 10.1084/jem.20041257 15657292 PMC2212798

[42] Hirota K Hashimoto M Yoshitomi H Tanaka S Nomura T Yamaguchi T T cell self-reactivity forms a cytokine milieu for spontaneous development of IL-17+ Th cells that cause autoimmune arthritis. J Exp Med (2007) 204:41–7 10.1084/jem.20062259 17227914 PMC2118414

[43] Fujino S Andoh A Bamba S Ogawa A Hata K Araki Y Increased expression of interleukin 17 in inflammatory bowel disease. Gut (2003) 52:65–70 10.1136/gut.52.1.65 12477762 PMC1773503

[44] Buhner S Buning C Genschel J Kling K Herrmann D Dignass A Genetic basis for increased intestinal permeability in families with Crohn’s disease: role of CARD15 3020insC mutation? Gut (2006) 55:342–7 10.1136/gut.2005.065557 16000642 PMC1856071

[45] D’Inca R Annese V di Leo V Latiano A Quaino V Abazia C Increased intestinal permeability and NOD2 variants in familial and sporadic Crohn’s disease. Aliment Pharmacol Ther (2006) 23:1455–61 10.1111/j.1365-2036.2006.02916.x 16669960

[46] Schreiber S Slipping the barrier: how variants in CARD15 could alter permeability of the intestinal wall and population health. Gut (2006) 55:308–9 10.1136/gut.2005.076075 16474103 PMC1856076

[47] Chamaillard M Hashimoto M Horie Y Masumoto J Qiu S Saab L An essential role for NOD1 in host recognition of bacterial peptidoglycan containing diaminopimelic acid. Nat Immunol (2003) 4:702–7 10.1038/ni945 12796777

[48] McGovern DP Hysi P Ahmad T van Heel DA Moffatt MF Carey A Association between a complex insertion/deletion polymorphism in NOD1 (CARD4) and susceptibility to inflammatory bowel disease. Hum Mol Genet (2005) 14:1245–50 10.1093/hmg/ddi135 15790594

[49] Rioux JD Xavier RJ Taylor KD Silverberg MS Goyette P Huett A Genome-wide association study identifies new susceptibility loci for Crohn disease and implicates autophagy in disease pathogenesis. Nat Genet (2007) 39:596–604 10.1038/ng2032 17435756 PMC2757939

[50] Parkes M Barrett JC Prescott NJ Tremelling M Anderson CA Fisher SA Sequence variants in the autophagy gene IRGM and multiple other replicating loci contribute to Crohn’s disease susceptibility. Nat Genet (2007) 39:830–2 10.1038/ng2061 17554261 PMC2628541

[51] Klionsky DJ Emr SD Autophagy as a regulated pathway of cellular degradation. Science (2000) 290:1717–21 10.1126/science.290.5497.1717 11099404 PMC2732363

[52] Burton PR Clayton DG Cardon LR Craddock N Deloukas P Duncanson A Association scan of 14,500 nonsynonymous SNPs in four diseases identifies autoimmunity variants. Nat Genet (2007) 39:1329–37 10.1038/ng.2007.17 17952073 PMC2680141

[53] Hampe J Franke A Rosenstiel P Till A Teuber M Huse K A genome-wide association scan of nonsynonymous SNPs identifies a susceptibility variant for Crohn disease in ATG16L1. Nat Genet (2007) 39:207–11 10.1038/ng1954 17200669

[54] Cadwell K Liu JY Brown SL Miyoshi H Loh J Lennerz JK A key role for autophagy and the autophagy gene Atg16l1 in mouse and human intestinal Paneth cells. Nature (2008) 456:259–63 10.1038/nature07416 18849966 PMC2695978

[55] Shorter RG Huizenga KA Spencer RJ A working hypothesis for the etiology and pathogenesis of nonspecific inflammatory bowel disease. Am J Dig Dis (1972) 17:1024–32 10.1007/BF02239143 5082428

[56] Cereijido M Contreras RG Flores-Benitez D Flores-Maldonado C Larre I Ruiz A New diseases derived or associated with the tight junction. Arch Med Res (2007) 38:465–78 10.1016/j.arcmed.2007.02.003 17560451

[57] Okayasu I Hatakeyama S Yamada M Ohkusa T Inagaki Y Nakaya R A novel method in the induction of reliable experimental acute and chronic ulcerative colitis in mice. Gastroenterology (1990) 98:694–702 1688816 10.1016/0016-5085(90)90290-h

[58] Poritz LS Garver KI Green C Fitzpatrick L Ruggiero F Koltun WA Loss of the tight junction protein ZO-1 in dextran sulfate sodium induced colitis. J Surg Res (2007) 140:12–9 10.1016/j.jss.2006.07.050 17418867

[59] Mahler M Bristol IJ Sundberg JP Churchill GA Birkenmeier EH Elson CO Genetic analysis of susceptibility to dextran sulfate sodium-induced colitis in mice. Genomics (1999) 55:147–56 10.1006/geno.1998.5636 9933561

[60] Dieleman LA Ridwan BU Tennyson GS Beagley KW Bucy RP Elson CO Dextran sulfate sodium-induced colitis occurs in severe combined immunodeficient mice. Gastroenterology (1994) 107:1643–52 7958674 10.1016/0016-5085(94)90803-6

[61] Morris GP Beck PL Herridge MS Depew WT Szewczuk MR Wallace JL Hapten-induced model of chronic inflammation and ulceration in the rat colon. Gastroenterology (1989) 96:795–803 2914642

[62] Hermiston ML Gordon JI Inflammatory bowel disease and adenomas in mice expressing a dominant negative N-cadherin. Science (1995) 270:1203–7 10.1126/science.270.5239.1203 7502046

[63] Smalley-Freed WG Efimov A Burnett PE Short SP Davis MA Gumucio DL p120-Catenin is essential for maintenance of barrier function and intestinal homeostasis in mice. J Clin Invest (2010) 120:1824–35 10.1172/JCI41414 20484816 PMC2877948

[64] Rudolph U Finegold MJ Rich SS Harriman GR Srinivasan Y Brabet P Gi2 alpha protein deficiency: a model of inflammatory bowel disease. J Clin Immunol (1995) 15:101S–5S 10.1007/BF01540899 8613481

[65] Saha C Nigam SK Denker BM Involvement of Galphai2 in the maintenance and biogenesis of epithelial cell tight junctions. J Biol Chem (1998) 273:21629–33 10.1074/jbc.273.34.21629 9705295

[66] Laukoetter MG Nava P Lee WY Severson EA Capaldo CT Babbin BA JAM-A regulates permeability and inflammation in the intestine in vivo. J Exp Med (2007) 204:3067–76 10.1084/jem.20071416 18039951 PMC2150975

[67] Woodfin A Reichel CA Khandoga A Corada M Voisin MB Scheiermann C JAM-A mediates neutrophil transmigration in a stimulus-specific manner in vivo: evidence for sequential roles for JAM-A and PECAM-1 in neutrophil transmigration. Blood (2007) 110:1848–56 10.1182/blood-2006-09-047431 17505016

[68] Cera MR Del Prete A Vecchi A Corada M Martin-Padura I Motoike T Increased DC trafficking to lymph nodes and contact hypersensitivity in junctional adhesion molecule-A-deficient mice. J Clin Invest (2004) 114:729–38 10.1172/JCI200421231 15343392 PMC514585

[69] Vetrano S Rescigno M Rosaria Cera M Correale C Rumio C Doni A Unique role of junctional adhesion molecule-a in maintaining mucosal homeostasis in inflammatory bowel disease. Gastroenterology (2008) 135:173–84 10.1053/j.gastro.2008.04.002 18514073

[70] Khounlotham M Kim W Peatman E Nava P Medina-Contreras O Addis C Compromised intestinal epithelial barrier induces adaptive immune compensation that protects from colitis. Immunity (2012) 37:563–73 10.1016/j.immuni.2012.06.017 22981539 PMC3564580

[71] Schulzke JD Gitter AH Mankertz J Spiegel S Seidler U Amasheh S Epithelial transport and barrier function in occludin-deficient mice. Biochim Biophys Acta (2005) 1669:34–42 10.1016/j.bbamem.2005.01.008 15842997

[72] Tamura A Kitano Y Hata M Katsuno T Moriwaki K Sasaki H Megaintestine in claudin-15-deficient mice. Gastroenterology (2008) 134:523–34 10.1053/j.gastro.2007.11.040 18242218

[73] Su L Shen L Clayburgh DR Nalle SC Sullivan EA Meddings JB Targeted epithelial tight junction dysfunction causes immune activation and contributes to development of experimental colitis. Gastroenterology (2009) 136:551–63 10.1053/j.gastro.2008.10.081 19027740 PMC2712351

[74] Kosiewicz MM Nast CC Krishnan A Rivera-Nieves J Moskaluk CA Matsumoto S Th1-type responses mediate spontaneous ileitis in a novel murine model of Crohn’s disease. J Clin Invest (2001) 107:695–702 10.1172/JCI10956 11254669 PMC208944

[75] Pizarro TT Pastorelli L Bamias G Garg RR Reuter BK Mercado JR SAMP1/YitFc mouse strain: a spontaneous model of Crohn’s disease-like ileitis. Inflamm Bowel Dis (2011) 17:2566–84 10.1002/ibd.21638 21557393 PMC3154989

[76] Rivera-Nieves J Bamias G Vidrich A Marini M Pizarro TT McDuffie MJ Emergence of perianal fistulizing disease in the SAMP1/YitFc mouse, a spontaneous model of chronic ileitis. Gastroenterology (2003) 124:972–82 10.1053/gast.2003.50148 12671894

[77] Vidrich A Buzan JM Barnes S Reuter BK Skaar K Ilo C Altered epithelial cell lineage allocation and global expansion of the crypt epithelial stem cell population are associated with ileitis in SAMP1/YitFc mice. Am J Pathol (2005) 166:1055–67 10.1016/S0002-9440(10)62326-7 15793286 PMC1602382

[78] Olson TS Reuter BK Scott KG Morris MA Wang XM Hancock LN The primary defect in experimental ileitis originates from a nonhematopoietic source. J Exp Med (2006) 203:541–52 10.1084/jem.20050407 16505137 PMC2118253

[79] Furuse M Furuse K Sasaki H Tsukita S Conversion of zonulae occludentes from tight to leaky strand type by introducing claudin-2 into Madin-Darby canine kidney I cells. J Cell Biol (2001) 153:263–72 10.1083/jcb.153.2.263 11309408 PMC2169456

[80] Kozaiwa K Sugawara K Smith MF Jr Carl V Yamschikov V Belyea B Identification of a quantitative trait locus for ileitis in a spontaneous mouse model of Crohn’s disease: SAMP1/YitFc. Gastroenterology (2003) 125:477–90 10.1016/S0016-5085(03)00876-X 12891551

[81] Reuter BK Pizarro TT Mechanisms of tight junction dysregulation in the SAMP1/YitFc model of Crohn’s disease-like ileitis. Ann N Y Acad Sci (2009) 1165:301–7 10.1111/j.1749-6632.2009.04035.x 19538320

[82] Hollander D Vadheim CM Brettholz E Petersen GM Delahunty T Rotter JI Increased intestinal permeability in patients with Crohn’s disease and their relatives. A possible etiologic factor. Ann Intern Med (1986) 105:883–5 10.7326/0003-4819-105-6-883 3777713

[83] Kucharzik T Walsh SV Chen J Parkos CA Nusrat A Neutrophil transmigration in inflammatory bowel disease is associated with differential expression of epithelial intercellular junction proteins. Am J Pathol (2001) 159:2001–9 10.1016/S0002-9440(10)63051-9 11733350 PMC1850599

[84] D’Inca R Di Leo V Corrao G Martines D D’Odorico A Mestriner C Intestinal permeability test as a predictor of clinical course in Crohn’s disease. Am J Gastroenterol (1999) 94:2956–60 10.1111/j.1572-0241.1999.01444.x 10520851

[85] Arnott ID Kingstone K Ghosh S Abnormal intestinal permeability predicts relapse in inactive Crohn disease. Scand J Gastroenterol (2000) 35:1163–9 10.1080/003655200750056637 11145287

[86] Prasad S Mingrino R Kaukinen K Hayes KL Powell RM Macdonald TT Inflammatory processes have differential effects on claudins 2, 3 and 4 in colonic epithelial cells. Lab Invest (2005) 85:1139–62 10.1038/labinvest.3700316 16007110

[87] Zeissig S Burgel N Gunzel D Richter J Mankertz J Wahnschaffe U Changes in expression and distribution of claudin 2, 5 and 8 lead to discontinuous tight junctions and barrier dysfunction in active Crohn’s disease. Gut (2007) 56:61–72 10.1136/gut.2006.094375 16822808 PMC1856677

[88] Ridyard AE Brown JK Rhind SM Else RW Simpson JW Miller HR Apical junction complex protein expression in the canine colon: differential expression of claudin-2 in the colonic mucosa in dogs with idiopathic colitis. J Histochem Cytochem (2007) 55:1049–58 10.1369/jhc.7A7211.2007 17595339

[89] Barrett JC Lee JC Lees CW Prescott NJ Anderson CA Phillips A Genome-wide association study of ulcerative colitis identifies three new susceptibility loci, including the HNF4A region. Nat Genet (2009) 41:1330–4 10.1038/ng.483 19915572 PMC2812019

[90] Battle MA Konopka G Parviz F Gaggl AL Yang C Sladek FM Hepatocyte nuclear factor 4alpha orchestrates expression of cell adhesion proteins during the epithelial transformation of the developing liver. Proc Natl Acad Sci U S A (2006) 103:8419–24 10.1073/pnas.0600246103 16714383 PMC1482507

[91] Garrison WD Battle MA Yang C Kaestner KH Sladek FM Duncan SA Hepatocyte nuclear factor 4alpha is essential for embryonic development of the mouse colon. Gastroenterology (2006) 130:1207–20 10.1053/j.gastro.2006.01.003 16618389 PMC3581272

[92] Ahn SH Shah YM Inoue J Morimura K Kim I Yim S Hepatocyte nuclear factor 4alpha in the intestinal epithelial cells protects against inflammatory bowel disease. Inflamm Bowel Dis (2008) 14:908–20 10.1002/ibd.20413 18338782 PMC2435391

[93] van Bodegraven AA Curley CR Hunt KA Monsuur AJ Linskens RK Onnie CM Genetic variation in myosin IXB is associated with ulcerative colitis. Gastroenterology (2006) 131:1768–74 10.1053/j.gastro.2006.09.011 17087940

[94] McGovern DP Taylor KD Landers C Derkowski C Dutridge D Dubinsky M MAGI2 genetic variation and inflammatory bowel disease. Inflamm Bowel Dis (2008) 15(1):75–83 10.1002/ibd.20611 18720471 PMC2614310

[95] Wapenaar MC Monsuur AJ van Bodegraven AA Weersma RK Bevova MR Linskens RK Associations with tight junction genes PARD3 and MAGI2 in Dutch patients point to a common barrier defect for coeliac disease and ulcerative colitis. Gut (2008) 57:463–7 10.1136/gut.2007.133132 17989107

[96] Post PL Tyska MJ O’Connell CB Johung K Hayward A Mooseker MS Myosin-IXb is a single-headed and processive motor. J Biol Chem (2002) 277:11679–83 10.1074/jbc.M111173200 11801597

[97] Monsuur AJ de Bakker PI Alizadeh BZ Zhernakova A Bevova MR Strengman E Myosin IXB variant increases the risk of celiac disease and points toward a primary intestinal barrier defect. Nat Genet (2005) 37:1341–4 10.1038/ng1680 16282976

[98] Takahashi D Hase K Kimura S Nakatsu F Ohmae M Mandai Y The epithelia-specific membrane trafficking factor AP-1B controls gut immune homeostasis in mice. Gastroenterology (2011) 141:621–32 10.1053/j.gastro.2011.04.056 21669204

[99] Obata Y Takahashi D Ebisawa M Kakiguchi K Yonemura S Jinnohara T Epithelial cell-intrinsic Notch signaling plays an essential role in the maintenance of gut immune homeostasis. J Immunol (2012) 188:2427–36 10.4049/jimmunol.1101128 22279105

[100] Kaser A Lee AH Franke A Glickman JN Zeissig S Tilg H XBP1 links ER stress to intestinal inflammation and confers genetic risk for human inflammatory bowel disease. Cell (2008) 134:743–56 10.1016/j.cell.2008.07.021 18775308 PMC2586148

[101] Garabedian EM Roberts LJ McNevin MS Gordon JI Examining the role of Paneth cells in the small intestine by lineage ablation in transgenic mice. J Biol Chem (1997) 272:23729–40 10.1074/jbc.272.38.23729 9295317

[102] Van Limbergen J Russell RK Nimmo ER Satsangi J The genetics of inflammatory bowel disease. Am J Gastroenterol (2007) 102:2820–31 10.1111/j.1572-0241.2007.01527.x 17894847

[103] Panwala CM Jones JC Viney JL A novel model of inflammatory bowel disease: mice deficient for the multiple drug resistance gene, mdr1a, spontaneously develop colitis. J Immunol (1998) 161:5733–44 9820555

[104] Resta-Lenert S Smitham J Barrett KE Epithelial dysfunction associated with the development of colitis in conventionally housed mdr1a-/- mice. Am J Physiol Gastrointest Liver Physiol (2005) 289:G153–62 10.1152/ajpgi.00395.2004 15774938

[105] Staley EM Schoeb TR Lorenz RG Differential susceptibility of P-glycoprotein deficient mice to colitis induction by environmental insults. Inflamm Bowel Dis (2009) 15:684–96 10.1002/ibd.20824 19067430 PMC2887754

[106] Collett A Higgs NB Gironella M Zeef LA Hayes A Salmo E Early molecular and functional changes in colonic epithelium that precede increased gut permeability during colitis development in mdr1a(-/-) mice. Inflamm Bowel Dis (2008) 14(5):620–31 10.1002/ibd.20375 18275070

[107] Brant SR Panhuysen CI Nicolae D Reddy DM Bonen DK Karaliukas R MDR1 Ala893 polymorphism is associated with inflammatory bowel disease. Am J Hum Genet (2003) 73:1282–92 10.1086/379927 14610718 PMC1180394

[108] Tamai I Ohashi R Nezu JI Sai Y Kobayashi D Oku A Molecular and functional characterization of organic cation/carnitine transporter family in mice. J Biol Chem (2000) 275:40064–72 10.1074/jbc.M005340200 11010964

[109] Peltekova VD Wintle RF Rubin LA Amos CI Huang Q Gu X Functional variants of OCTN cation transporter genes are associated with Crohn disease. Nat Genet (2004) 36:471–5 10.1038/ng1339 15107849

[110] Stoll M Corneliussen B Costello CM Waetzig GH Mellgard B Koch WA Genetic variation in DLG5 is associated with inflammatory bowel disease. Nat Genet (2004) 36:476–80 10.1038/ng1345 15107852

[111] Nakamura H Sudo T Tsuiki H Miyake H Morisaki T Sasaki J Identification of a novel human homolog of the *Drosophila* dlg, P-dlg, specifically expressed in the gland tissues and interacting with p55. FEBS Lett (1998) 433:63–7 10.1016/S0014-5793(98)00882-5 9738934

[112] Festen EA Zhernakova A Wijmenga C Weersma RK Association of DLG5 variants with gluten-sensitive enteropathy. Gut (2008) 57:1027–8 10.1136/gut.2007.144857 18559397

[113] Friedrichs F Henckaerts L Vermeire S Kucharzik T Seehafer T Moller-Krull M The Crohn’s disease susceptibility gene DLG5 as a member of the CARD interaction network. J Mol Med (2008) 86:423–32 10.1007/s00109-008-0307-5 18335190

[114] Biank V Friedrichs F Babusukumar U Wang T Stoll M Broeckel U DLG5 R30Q variant is a female-specific protective factor in pediatric onset Crohn’s disease. Am J Gastroenterol (2007) 102:391–8 10.1111/j.1572-0241.2006.01011.x 17156146

[115] Browning BL Annese V Barclay ML Bingham SA Brand S Buning C Gender-stratified analysis of DLG5 R30Q in 4707 patients with Crohn disease and 4973 controls from 12 Caucasian cohorts. J Med Genet (2008) 45:36–42 10.1136/jmg.2007.050773 17693570

[116] Gibson P Rosella O Nov R Young G Colonic epithelium is diffusely abnormal in ulcerative colitis and colorectal cancer. Gut (1995) 36:857–63 10.1136/gut.36.6.857 7615274 PMC1382623

[117] Brandtzaeg P Molecular and cellular aspects of the secretory immunoglobulin system. APMIS (1995) 103:1–19 10.1111/j.1699-0463.1995.tb01073.x 7695886

[118] Weinberg ED Human lactoferrin: a novel therapeutic with broad spectrum potential. J Pharm Pharmacol (2001) 53:1303–10 10.1211/0022357011777792 11697537

[119] Proud D Kaplan AP Kinin formation: mechanisms and role in inflammatory disorders. Annu Rev Immunol (1988) 6:49–83 10.1146/annurev.iy.06.040188.000405 3289575

[120] Chao J Schmaier A Chen LM Yang Z Chao L Kallistatin, a novel human tissue kallikrein inhibitor: levels in body fluids, blood cells, and tissues in health and disease. J Lab Clin Med (1996) 127:612–20 10.1016/S0022-2143(96)90152-3 8648266

[121] Devani M Cugno M Vecchi M Ferrero S Di Berardino F Avesani EC Kallikrein-kinin system activation in Crohn’s disease: differences in intestinal and systemic markers. Am J Gastroenterol (2002) 97:2026–32 10.1111/j.1572-0241.2002.05919.x 12190172

[122] Devani M Vecchi M Ferrero S Avesani EC Arizzi C Chao L Kallikrein-kinin system in inflammatory bowel diseases: intestinal involvement and correlation with the degree of tissue inflammation. Dig Liver Dis (2005) 37:665–73 10.1016/j.dld.2005.01.021 15949977

[123] Van der Sluis M De Koning BA De Bruijn AC Velcich A Meijerink JP Van Goudoever JB Muc2-deficient mice spontaneously develop colitis, indicating that MUC2 is critical for colonic protection. Gastroenterology (2006) 131:117–29 10.1053/j.gastro.2006.04.020 16831596

[124] Heazlewood CK Cook MC Eri R Price GR Tauro SB Taupin D Aberrant mucin assembly in mice causes endoplasmic reticulum stress and spontaneous inflammation resembling ulcerative colitis. PLoS Med (2008) 5:e54 10.1371/journal.pmed.0050054 18318598 PMC2270292

[125] Einerhand AW Renes IB Makkink MK van der Sluis M Buller HA Dekker J Role of mucins in inflammatory bowel disease: important lessons from experimental models. Eur J Gastroenterol Hepatol (2002) 14:757–65 10.1097/00042737-200207000-00008 12169985

[126] Kyo K Parkes M Takei Y Nishimori H Vyas P Satsangi J Association of ulcerative colitis with rare VNTR alleles of the human intestinal mucin gene, MUC3. Hum Mol Genet (1999) 8:307–11 10.1093/hmg/8.2.307 9931338

[127] Kyo K Muto T Nagawa H Lathrop GM Nakamura Y Associations of distinct variants of the intestinal mucin gene MUC3A with ulcerative colitis and Crohn’s disease. J Hum Genet (2001) 46:5–20 10.1007/s100380170118 11289722

[128] Guilmeau S Flandez M Bancroft L Sellers RS Tear B Stanley P Intestinal deletion of Pofut1 in the mouse inactivates notch signaling and causes enterocolitis. Gastroenterology (2008) 135:e1–6 10.1053/j.gastro.2008.05.050 18621050 PMC3207497

[129] Bresso F Askling J Astegiano M Demarchi B Sapone N Rizzetto M Potential role for the common cystic fibrosis DeltaF508 mutation in Crohn’s disease. Inflamm Bowel Dis (2007) 13:531–6 10.1002/ibd.20067 17206681

[130] Korzenik JR Dieckgraefe BK Valentine JF Hausman DF Gilbert MJ Sargramostim for active Crohn’s disease. N Engl J Med (2005) 352:2193–201 10.1056/NEJMoa041109 15917384

[131] Unal AE Cevikel MH Ozgun H Tunger A Effect of granulocyte-macrophage colony stimulating factor on bacterial translocation after experimental obstructive jaundice. Eur J Surg (2001) 167:366–70 10.1080/110241501750215267 11419553

[132] Kurt-Jones EA Mandell L Whitney C Padgett A Gosselin K Newburger PE Role of toll-like receptor 2 (TLR2) in neutrophil activation: GM-CSF enhances TLR2 expression and TLR2-mediated interleukin 8 responses in neutrophils. Blood (2002) 100:1860–8 12176910

[133] Armitage JO Emerging applications of recombinant human granulocyte-macrophage colony-stimulating factor. Blood (1998) 92:4491–508 9845514

[134] Ramsay RG Micallef SJ Williams B Lightowler S Vincan E Heath JK Colony-stimulating factor-1 promotes clonogenic growth of normal murine colonic crypt epithelial cells in vitro. J Interferon Cytokine Res (2004) 24:416–27 10.1089/1079990041535638 15296653

[135] Pagnini C Cominelli F Probiotics in experimental and human inflammatory bowel disease: discussion points. Dig Liver Dis (2006) 38(Suppl 2):S270–3 10.1016/S1590-8658(07)60008-5 17259089

[136] Vanderpool C Yan F Polk DB Mechanisms of probiotic action: implications for therapeutic applications in inflammatory bowel diseases. Inflamm Bowel Dis (2008) 14(11):1585–96 10.1002/ibd.20525 18623173

[137] Dogi CA Galdeano CM Perdigon G Gut immune stimulation by non pathogenic Gram(+) and Gram(-) bacteria. Comparison with a probiotic strain. Cytokine (2008) 41:223–31 10.1016/j.cyto.2007.11.014 18248820

[138] Wehkamp J Harder J Wehkamp K Wehkamp-von Meissner B Schlee M Enders C NF-kappaB- and AP-1-mediated induction of human beta defensin-2 in intestinal epithelial cells by *Escherichia coli* Nissle 1917: a novel effect of a probiotic bacterium. Infect Immun (2004) 72:5750–8 10.1128/IAI.72.10.5750-5758.2004 15385474 PMC517557

[139] Grabig A Paclik D Guzy C Dankof A Baumgart DC Erckenbrecht J *Escherichia coli* strain Nissle 1917 ameliorates experimental colitis via toll-like receptor 2- and toll-like receptor 4-dependent pathways. Infect Immun (2006) 74:4075–82 10.1128/IAI.01449-05 16790781 PMC1489743

[140] Bai AP Ouyang Q Probiotics and inflammatory bowel diseases. Postgrad Med J (2006) 82:376–82 10.1136/pgmj.2005.040899 16754706 PMC2563748

[141] Bernet MF Brassart D Neeser JR Servin AL *Lactobacillus acidophilus* LA 1 binds to cultured human intestinal cell lines and inhibits cell attachment and cell invasion by enterovirulent bacteria. Gut (1994) 35:483–9 10.1136/gut.35.4.483 8174985 PMC1374796

[142] Schultz M Scholmerich J Rath HC Rationale for probiotic and antibiotic treatment strategies in inflammatory bowel diseases. Dig Dis (2003) 21:105–28 10.1159/000073243 14571109

[143] Paterson BM Lammers KM Arrieta MC Fasano A Meddings JB The safety, tolerance, pharmacokinetic and pharmacodynamic effects of single doses of AT-1001 in coeliac disease subjects: a proof of concept study. Aliment Pharmacol Ther (2007) 26:757–66 10.1111/j.1365-2036.2007.03413.x 17697209

[144] Arrieta MC Madsen K Doyle J Meddings J Reducing small intestinal permeability attenuates colitis in the IL10 gene-deficient mouse. Gut (2009) 58:41–8 10.1136/gut.2008.150888 18829978 PMC2597688

[145] Leffler DA Kelly CP Abdallah HZ Colatrella AM Harris LA Leon F A randomized, double-blind study of larazotide acetate to prevent the activation of celiac disease during gluten challenge. Am J Gastroenterol (2012) 107:1554–62 10.1038/ajg.2012.211 22825365 PMC3463856

[146] Kong J Zhang Z Musch MW Ning G Sun J Hart J Novel role of the vitamin D receptor in maintaining the integrity of the intestinal mucosal barrier. Am J Physiol Gastrointest Liver Physiol (2008) 294:G208–16 10.1152/ajpgi.00398.2007 17962355

[147] Palmer HG Gonzalez-Sancho JM Espada J Berciano MT Puig I Baulida J Vitamin D(3) promotes the differentiation of colon carcinoma cells by the induction of E-cadherin and the inhibition of beta-catenin signaling. J Cell Biol (2001) 154:369–87 10.1083/jcb.200102028 11470825 PMC2150773

[148] Simmons JD Mullighan C Welsh KI Jewell DP Vitamin D receptor gene polymorphism: association with Crohn’s disease susceptibility. Gut (2000) 47:211–4 10.1136/gut.47.2.211 10896912 PMC1728007

[149] Dresner-Pollak R Ackerman Z Eliakim R Karban A Chowers Y Fidder HH The BsmI vitamin D receptor gene polymorphism is associated with ulcerative colitis in Jewish Ashkenazi patients. Genet Test (2004) 8:417–20 10.1089/gte.2004.8.417 15684874

[150] Whitacre CC Reingold SC O’Looney PA A gender gap in autoimmunity. Science (1999) 283:1277–8 10.1126/science.283.5406.1277 10084932

[151] Saibeni S Cortinovis I Beretta L Tatarella M Ferraris L Rondonotti E Gender and disease activity influence health-related quality of life in inflammatory bowel diseases. Hepatogastroenterology (2005) 52:509–15 15816468

[152] Pelissier MA Muller C Hill M Morfin R Protection against dextran sodium sulfate-induced colitis by dehydroepiandrosterone and 7alpha-hydroxy-dehydroepiandrosterone in the rat. Steroids (2006) 71:240–8 10.1016/j.steroids.2005.10.009 16371229

[153] Harnish DC Albert LM Leathurby Y Eckert AM Ciarletta A Kasaian M Beneficial effects of estrogen treatment in the HLA-B27 transgenic rat model of inflammatory bowel disease. Am J Physiol Gastrointest Liver Physiol (2004) 286:G118–25 10.1152/ajpgi.00024.2003 12958017

[154] Braniste V Leveque M Buisson-Brenac C Bueno L Fioramonti J Houdeau E Oestradiol decreases colonic permeability through oestrogen receptor beta-mediated up-regulation of occludin and junctional adhesion molecule-A in epithelial cells. J Physiol (2009) 587:3317–28 10.1113/jphysiol.2009.169300 19433574 PMC2727039

[155] Shen J Ran ZH Tong JL Xiao SD Meta-analysis: the utility and safety of heparin in the treatment of active ulcerative colitis. Aliment Pharmacol Ther (2007) 26:653–63 10.1111/j.1365-2036.2007.03418.x 17697199

[156] Celasco G Moro L Bozzella R Mangano K Quattrocchi C Aiello C Efficacy of intracolonic administration of low-molecular-weight heparin CB-01-05, compared to other low-molecular-weight heparins and unfractionated heparin, in experimentally induced colitis in rat. Dig Dis Sci (2008) 53(12):3170–5 10.1007/s10620-008-0299-6 18465235

[157] Pellequer Y Meissner Y Ubrich N Lamprecht A Epithelial heparin delivery via microspheres mitigates experimental colitis in mice. J Pharmacol Exp Ther (2007) 321:726–33 10.1124/jpet.106.117226 17322027

[158] Pastorelli L Saibeni S Spina L Signorelli C Celasco G de Franchis R Oral, colonic-release low-molecular-weight heparin: an initial open study of Parnaparin-MMX for the treatment of mild-to-moderate left-sided ulcerative colitis. Aliment Pharmacol Ther (2008) 28:581–8 10.1111/j.1365-2036.2008.03757.x 18700898

[159] Celasco G Papa A Jones R Moro L Bozzella R Surace MM Clinical trial: oral colon-release parnaparin sodium tablets (CB-01-05 MMX) for active left-sided ulcerative colitis. Aliment Pharmacol Ther (2010) 31:375–86 10.1111/j.1365-2036.2009.04194.x 19891665

[160] Murch SH Macdonald TT Walker-Smith JA Levin M Lionetti P Klein NJ Disruption of sulphated glycosaminoglycans in intestinal inflammation. Lancet (1993) 341:711–4 10.1016/0140-6736(93)90485-Y 8095623

[161] Murch SH Winyard PJ Koletzko S Wehner B Cheema HA Risdon RA Congenital enterocyte heparan sulphate deficiency with massive albumin loss, secretory diarrhoea, and malnutrition. Lancet (1996) 347:1299–301 10.1016/S0140-6736(96)90941-1 8622507

[162] Westphal V Murch S Kim S Srikrishna G Winchester B Day R Reduced heparan sulfate accumulation in enterocytes contributes to protein-losing enteropathy in a congenital disorder of glycosylation. Am J Pathol (2000) 157:1917–25 10.1016/S0002-9440(10)64830-4 11106564 PMC1885788

[163] Bode L Salvestrini C Park PW Li JP Esko JD Yamaguchi Y Heparan sulfate and syndecan-1 are essential in maintaining murine and human intestinal epithelial barrier function. J Clin Invest (2008) 118:229–38 10.1172/JCI32335 18064305 PMC2117765

[164] Commane DM Shortt CT Silvi S Cresci A Hughes RM Rowland IR Effects of fermentation products of pro- and prebiotics on trans-epithelial electrical resistance in an in vitro model of the colon. Nutr Cancer (2005) 51:102–9 10.1207/s15327914nc5101_14 15749636

[165] Qin HL Shen TY Gao ZG Fan XB Hang XM Jiang YQ Effect of *Lactobacillus* on the gut microflora and barrier function of the rats with abdominal infection. World J Gastroenterol (2005) 11:2591–6 15849817 10.3748/wjg.v11.i17.2591 PMC4305749

[166] Zyrek AA Cichon C Helms S Enders C Sonnenborn U Schmidt MA Molecular mechanisms underlying the probiotic effects of *Escherichia coli* Nissle 1917 involve ZO-2 and PKCzeta redistribution resulting in tight junction and epithelial barrier repair. Cell Microbiol (2007) 9:804–16 10.1111/j.1462-5822.2006.00836.x 17087734

[167] Mennigen R Nolte K Rijcken E Utech M Loeffler B Senninger N Probiotic mixture VSL#3 protects the epithelial barrier by maintaining tight junction protein expression and preventing apoptosis in a murine model of colitis. Am J Physiol Gastrointest Liver Physiol (2009) 296:G1140–9 10.1152/ajpgi.90534.2008 19221015

[168] Corridoni D Pastorelli L Mattioli B Locovei S Ishikawa D Arseneau KO Probiotic bacteria regulate intestinal epithelial permeability in experimental ileitis by a TNF-dependent mechanism. PLoS One (2012) 7:e42067 10.1371/journal.pone.0042067 22848704 PMC3405026

[169] Pagnini C Saeed R Bamias G Arseneau KO Pizarro TT Cominelli F Probiotics promote gut health through stimulation of epithelial innate immunity. Proc Natl Acad Sci U S A (2010) 107:454–9 10.1073/pnas.0910307107 20018654 PMC2806692

[170] Podolsky DK Fournier DA Alterations in mucosal content of colonic glycoconjugates in inflammatory bowel disease defined by monoclonal antibodies. Gastroenterology (1988) 95:379–87 3292335 10.1016/0016-5085(88)90494-5

[171] Ardizzone S Bianchi Porro G Biologic therapy for inflammatory bowel disease. Drugs (2005) 65:2253–86 10.2165/00003495-200565160-00002 16266194

[172] Suenaert P Bulteel V Lemmens L Noman M Geypens B Van Assche G Anti-tumor necrosis factor treatment restores the gut barrier in Crohn’s disease. Am J Gastroenterol (2002) 97:2000–4 10.1111/j.1572-0241.2002.05914.x 12190167

[173] Suenaert P Bulteel V Vermeire S Noman M Van Assche G Rutgeerts P Hyperresponsiveness of the mucosal barrier in Crohn’s disease is not tumor necrosis factor-dependent. Inflamm Bowel Dis (2005) 11:667–73 10.1097/01.MIB.0000168371.87283.4b 15973122

[174] Zeissig S Bojarski C Buergel N Mankertz J Zeitz M Fromm M Downregulation of epithelial apoptosis and barrier repair in active Crohn’s disease by tumour necrosis factor alpha antibody treatment. Gut (2004) 53:1295–302 10.1136/gut.2003.036632 15306588 PMC1774168

[175] Marini M Bamias G Rivera-Nieves J Moskaluk CA Hoang SB Ross WG TNF-alpha neutralization ameliorates the severity of murine Crohn’s-like ileitis by abrogation of intestinal epithelial cell apoptosis. Proc Natl Acad Sci U S A (2003) 100:8366–71 10.1073/pnas.1432897100 12832622 PMC166235

[176] Mirpuri J Brazil JC Berardinelli AJ Nasr TR Cooper K Schnoor M Commensal *Escherichia coli* reduces epithelial apoptosis through IFN-alphaA-mediated induction of guanylate binding protein-1 in human and murine models of developing intestine. J Immunol (2010) 184:7186–95 10.4049/jimmunol.0903116 20483731 PMC3145319

[177] Sinha A Nightingale J West KP Berlanga-Acosta J Playford RJ Epidermal growth factor enemas with oral mesalamine for mild-to-moderate left-sided ulcerative colitis or proctitis. N Engl J Med (2003) 349:350–7 10.1056/NEJMoa013136 12878742

[178] Mashimo H Wu DC Podolsky DK Fishman MC Impaired defense of intestinal mucosa in mice lacking intestinal trefoil factor. Science (1996) 274:262–5 10.1126/science.274.5285.262 8824194

[179] Mahmood A Melley L Fitzgerald AJ Ghosh S Playford RJ Trial of trefoil factor 3 enemas, in combination with oral 5-aminosalicylic acid, for the treatment of mild-to-moderate left-sided ulcerative colitis. Aliment Pharmacol Ther (2005) 21:1357–64 10.1111/j.1365-2036.2005.02436.x 15932366

[180] Bock J Liebisch G Schweimer J Schmitz G Rogler G Exogenous sphingomyelinase causes impaired intestinal epithelial barrier function. World J Gastroenterol (2007) 13:5217–25 17876892 10.3748/wjg.v13.i39.5217 PMC4171303

[181] Gulbins E Kolesnick R Raft ceramide in molecular medicine. Oncogene (2003) 22:7070–7 10.1038/sj.onc.1207146 14557812

[182] Megha S London E Ceramide selectively displaces cholesterol from ordered lipid domains (rafts): implications for lipid raft structure and function. J Biol Chem (2004) 279:9997–10004 14699154 10.1074/jbc.M309992200

[183] Rosella O Sinclair A Gibson PR Polyunsaturated fatty acids reduce non-receptor-mediated transcellular permeation of protein across a model of intestinal epithelium in vitro. J Gastroenterol Hepatol (2000) 15:626–31 10.1046/j.1440-1746.2000.02215.x 10921416

[184] Teitelbaum JE Allan Walker W Review: the role of omega 3 fatty acids in intestinal inflammation. J Nutr Biochem (2001) 12:21–32 10.1016/S0955-2863(00)00141-8 11179858

[185] Usami M Komurasaki T Hanada A Kinoshita K Ohata A Effect of gamma-linolenic acid or docosahexaenoic acid on tight junction permeability in intestinal monolayer cells and their mechanism by protein kinase C activation and/or eicosanoid formation. Nutrition (2003) 19:150–6 10.1016/S0899-9007(02)00927-9 12591548

[186] Aslan A Triadafilopoulos G Fish oil fatty acid supplementation in active ulcerative colitis: a double-blind, placebo-controlled, crossover study. Am J Gastroenterol (1992) 87:432–7 1553930

[187] Belluzzi A Brignola C Campieri M Pera A Boschi S Miglioli M Effect of an enteric-coated fish-oil preparation on relapses in Crohn’s disease. N Engl J Med (1996) 334:1557–60 10.1056/NEJM199606133342401 8628335

[188] Lorenz-Meyer H Bauer P Nicolay C Schulz B Purrmann J Fleig WE Omega-3 fatty acids and low carbohydrate diet for maintenance of remission in Crohn’s disease. A randomized controlled multicenter trial. Study Group Members (German Crohn’s Disease Study Group). Scand J Gastroenterol (1996) 31:778–85 10.3109/00365529609010352 8858747

[189] MacLean CH Mojica WA Newberry SJ Pencharz J Garland RH Tu W Systematic review of the effects of n-3 fatty acids in inflammatory bowel disease. Am J Clin Nutr (2005) 82:611–9 16155275 10.1093/ajcn.82.3.611

[190] Stremmel W Merle U Zahn A Autschbach F Hinz U Ehehalt R Retarded release phosphatidylcholine benefits patients with chronic active ulcerative colitis. Gut (2005) 54:966–71 10.1136/gut.2004.052316 15951544 PMC1774598

[191] Stremmel W Ehehalt R Autschbach F Karner M Phosphatidylcholine for steroid-refractory chronic ulcerative colitis: a randomized trial. Ann Intern Med (2007) 147:603–10 10.7326/0003-4819-147-9-200711060-00004 17975182

[192] Ehehalt R Wagenblast J Erben G Lehmann WD Hinz U Merle U Phosphatidylcholine and lysophosphatidylcholine in intestinal mucus of ulcerative colitis patients. A quantitative approach by nanoElectrospray-tandem mass spectrometry. Scand J Gastroenterol (2004) 39:737–42 10.1080/00365520410006233 15513358

[193] Tai EK Wong HP Lam EK Wu WK Yu L Koo MW Cathelicidin stimulates colonic mucus synthesis by up-regulating MUC1 and MUC2 expression through a mitogen-activated protein kinase pathway. J Cell Biochem (2008) 104:251–8 10.1002/jcb.21615 18059019

[194] Finnie IA Dwarakanath AD Taylor BA Rhodes JM Colonic mucin synthesis is increased by sodium butyrate. Gut (1995) 36:93–9 10.1136/gut.36.1.93 7890244 PMC1382360

[195] Hatayama H Iwashita J Kuwajima A Abe T The short chain fatty acid, butyrate, stimulates MUC2 mucin production in the human colon cancer cell line, LS174T. Biochem Biophys Res Commun (2007) 356:599–603 10.1016/j.bbrc.2007.03.025 17374366

[196] Barcelo A Claustre J Moro F Chayvialle JA Cuber JC Plaisancie P Mucin secretion is modulated by luminal factors in the isolated vascularly perfused rat colon. Gut (2000) 46:218–24 10.1136/gut.46.2.218 10644316 PMC1727811

[197] Mariadason JM Barkla DH Gibson PR Effect of short-chain fatty acids on paracellular permeability in Caco-2 intestinal epithelium model. Am J Physiol (1997) 272:G705–12 9142899 10.1152/ajpgi.1997.272.4.G705

[198] Peng L He Z Chen W Holzman IR Lin J Effects of butyrate on intestinal barrier function in a Caco-2 cell monolayer model of intestinal barrier. Pediatr Res (2007) 61:37–41 10.1203/01.pdr.0000250014.92242.f3 17211138

[199] Mariadason JM Kilias D Catto-Smith A Gibson PR Effect of butyrate on paracellular permeability in rat distal colonic mucosa ex vivo. J Gastroenterol Hepatol (1999) 14:873–9 10.1046/j.1440-1746.1999.01972.x 10535468

[200] Hamer HM Jonkers D Venema K Vanhoutvin S Troost FJ Brummer RJ Review article: the role of butyrate on colonic function. Aliment Pharmacol Ther (2008) 27:104–19 10.1111/j.1365-2036.2007.03562.x 17973645

[201] Steinhart AH Hiruki T Brzezinski A Baker JP Treatment of left-sided ulcerative colitis with butyrate enemas: a controlled trial. Aliment Pharmacol Ther (1996) 10:729–36 10.1046/j.1365-2036.1996.d01-509.x 8899080

[202] Vernia P Annese V Bresci G D’Albasio G D’Inca R Giaccari S Topical butyrate improves efficacy of 5-ASA in refractory distal ulcerative colitis: results of a multicentre trial. Eur J Clin Invest (2003) 33:244–8 10.1046/j.1365-2362.2003.01130.x 12641543

